# Cell-type-specific population dynamics of diverse reward computations

**DOI:** 10.1016/j.cell.2022.08.019

**Published:** 2022-09-15

**Authors:** Emily L. Sylwestrak, YoungJu Jo, Sam Vesuna, Xiao Wang, Blake Holcomb, Rebecca H. Tien, Doo Kyung Kim, Lief Fenno, Charu Ramakrishnan, William E. Allen, Ritchie Chen, Krishna V. Shenoy, David Sussillo, Karl Deisseroth

**Affiliations:** 1 Department of Biology, University of Oregon, Eugene, OR 97403, USA; 2 Department of Bioengineering, Stanford University, Stanford, CA 94305, USA; 3 Department of Applied Physics, Stanford University, Stanford, CA 94305, USA; 4 Department of Psychiatry and Behavioral Sciences, Stanford University, Stanford, CA 94305, USA; 5 Institute of Neuroscience, University of Oregon, Eugene, OR 97403, USA; 6 Neurosciences Interdepartmental Program, Stanford University, Stanford, CA 94303, USA; 7 Department of Neurobiology, Stanford University, Stanford, CA 94303, USA; 8 Department of Electrical Engineering, Stanford University, Stanford, CA, USA; 9 Wu Tsai Neurosciences Institute, Stanford University, Stanford, CA, USA; 10 Howard Hughes Medical Institute, Stanford University, Stanford, CA 94305, USA; 11 These authors contributed equally; 12 Lead contact

## Abstract

Computational analysis of cellular activity has developed largely independently of modern transcriptomic cell typology, but integrating these approaches may be essential for full insight into cellular-level mechanisms underlying brain function and dysfunction. Applying this approach to the habenula (a structure with diverse, intermingled molecular, anatomical, and computational features), we identified encoding of reward-predictive cues and reward outcomes in distinct genetically defined neural populations, including TH^+^ cells and Tac1^+^ cells. Data from genetically targeted recordings were used to train an optimized nonlinear dynamical systems model and revealed activity dynamics consistent with a line attractor. High-density, cell-type-specific electrophysiological recordings and optogenetic perturbation provided supporting evidence for this model. Reverse-engineering predicted how Tac1^+^ cells might integrate reward history, which was complemented by *in vivo* experimentation. This integrated approach describes a process by which data-driven computational models of population activity can generate and frame actionable hypotheses for cell-type-specific investigation in biological systems.

## In brief

Data-driven computational models of large-scale neural recordings predict celltype-specific probability dependence of reward response.

## Graphical Abstract

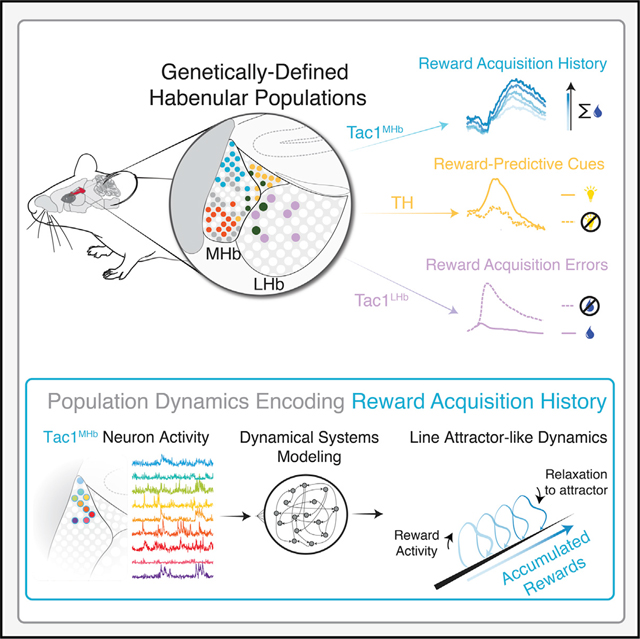

## INTRODUCTION

Across the animal kingdom, genetic targeting of cells within intermingled populations has enabled neuroscientists to identify specific cell types that can encode and drive precise brain states and behaviors (Adamantidis et al., 2007; Allen et al., 2019; Andalman et al., 2019; Buchholtz et al., 2020; Deisseroth, 2017; Füzesi et al., 2016; Jennings et al., 2019; Kim et al., 2013; Kohl et al., 2018; Krashes et al., 2014; Lovett-Barron et al., 2017, 2020; Sternson, 2020; Vesuna et al., 2020; Yu et al., 2009). Functional cell typology discoveries have generally not incorporated advances in neural population dynamics (in part because these analyses often use electrophysiology data, from which cell type identification is challenging to obtain). Yet understanding how molecularly defined populations are involved in brain and behavioral states may benefit from, or even require, integration of modern computational approaches.

Even in highly trained and well-performed behavior, cellular resolution brain activity exhibits substantial trial-to-trial variability that is often not well described by traditional concepts such as trial-averaged representations (Afshar et al., 2011; Pandarinath et al., 2018a; Yu et al., 2009; Mazzucato, 2022); similarly, under identical stimulus conditions, neural perturbations can elicit diverse neural and behavioral effects (Hallam, 1981; Pandarinath et al., 2018a; Remington et al., 2018; Shenoy et al., 2013; Sohn et al., 2019). Key conceptual advances have resulted from applying dynamical systems theory (e.g., Churchland et al., 2010; Shenoy et al., 2013); this approach, compared to the trial-averaged representational approach, may be of particular relevance for insight into neural and behavioral processes for which trial-to-trial variability itself has structure (for example, neural activity increasing as animals gather rewards during foraging in a time-varying environment).

To integrate genetic methods with computational methods leveraging population activity, we chose to study the transcriptionally diverse habenula, an epithalamic structure containing two main subdivisions with distinct gene expression, cytoarchitecture, and connectivity (Aizawa et al., 2012; Hikosaka, 2010; Namboodiri et al., 2016; Wagner et al., 2016). The lateral habenula (LHb) receives input from basal forebrain, prefrontal cortex, hypothalamus, and basal ganglia (Herkenham and Nauta, 1977; Hong and Hikosaka, 2008; Lazaridis et al., 2019; Stamatakis et al., 2013; Zhang et al., 2018) and sends projections to the ventral tegmental area (VTA/RMTg) and the dorsal raphe (Hong et al., 2011; Morales and Margolis, 2017; Quina et al., 2015). In contrast, the medial habenula (MHb) receives most of its input from the septum and nucleus of the diagonal band (Qin and Luo, 2009; Sutherland, 1982) and projects almost exclusively to the interpeduncular nucleus (IPN) (Qin and Luo, 2009; Quina et al., 2015). The relative simplicity of this anatomy belies its molecular diversity (Aizawa et al., 2012; Wagner et al., 2016; Díaz et al., 2011; Pandey et al., 2018). Classical habenula studies have focused on two mutually exclusive populations: dorsal tachykinin 1-expressing (Tac1, peptidergic) and ventral choline acetyltransferase-expressing (ChAT, cholinergic) neurons (Contestabile and Fonnum, 1983). Studies leveraging these genes have provided evidence for molecular-functional mapping, including the role of ChAT^+^ neurons in anxiety, nicotine sensitivity, and fear, as well as recent work identifying a role for Tac1^+^ neurons in spatial learning and motivated behavior (Cho et al., 2019; Seigneur et al., 2018; Yamaguchi et al., 2013; Pang et al., 2016; Frahm et al., 2011; Zhao-Shea et al., 2015; Hsu et al., 2014). Identification of additional cell type markers might further refine this mapping of cell type to function, particularly when extending the definition of function to the dynamical regime.

Here, we integrate spatially resolved transcript amplicon readout mapping (STARmap) (Kebschull et al., 2020; Wang et al., 2018) with genetically resolved optical and electrical recording to link specific cellular populations to behavioral elements of reward seeking. We show that habenular tyrosine hydroxylase-expressing (TH^+^) neurons learn and encode reward-predicting cues, LHb Tac1^+^ neurons encode negative reward outcomes, and MHb Tac1^+^ neurons integrate rewards with accumulation dynamics that are well described by a line attractor. Using an approach custom-modified for Ca^2+^ signals, we demonstrate nonlinear dynamical systems modeling of *in silico* behavioral sessions to computationally test alternative reward contingencies, and finally compare the model’s activity dynamics to experimentally measured dynamics in mice. Together, this approach illustrates the combination of spatial and genetic cell typology information with dynamical systems computational modeling for elucidating the functional significance of neural populations in behaving animals.

## RESULTS

### Molecular characterization of the habenula

**Figure 1.**
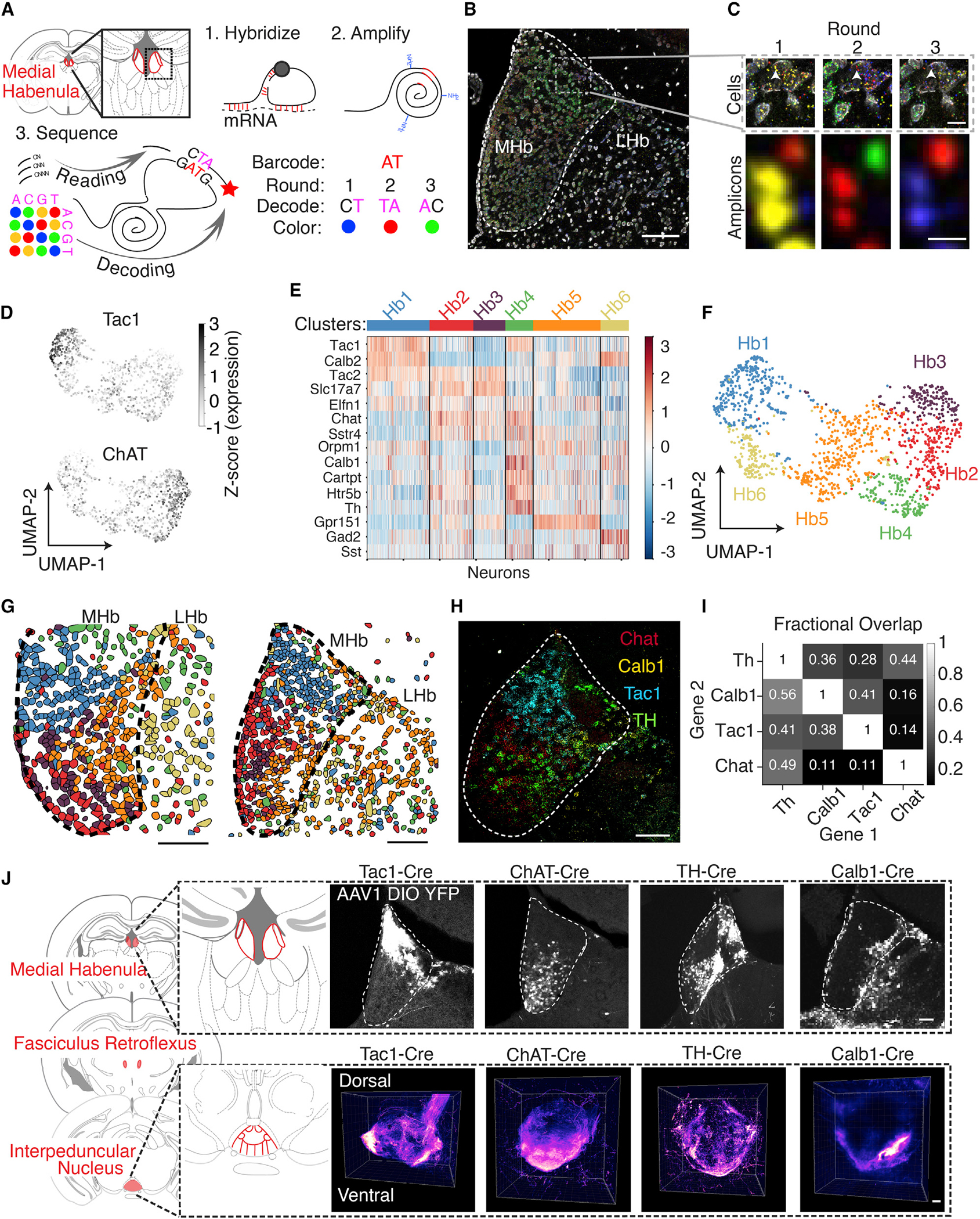
Molecular and anatomical characterization of medial habenula cell types. (A) Experimental design for 3 rounds of STARmap *in situ* sequencing of 15 genes in the habenula (Hb). Barcoded probes hybridize to mRNA targets and undergo rolling circle amplification. Sequential hybridization decodes each base on two adjacent rounds (STAR Methods). (B) Deconvolved image from one round of *in situ* sequencing of Hb tissue. Dashed line indicates MHb boundaries. Box indicates ROI in (C). Scalebar: 100 μm. (C) Top, magnified view of dotted box in (B) across 3 rounds of imaging. Scale bar: 10 μm. Bottom, magnified view indicated by arrow in top panels. Scale bar: 1 μm. (D) Uniform manifold approximation projection (UMAP) of the expression of 15 genes for 1440 segmented Hb neurons from 2 biological replicates. Grayscale indicates the *Z* scored expression of *Tac1* and *Chat*. (E) Heatmap of expression levels of each gene (row) for each cell (column), color bar indicates *Z* score for each gene across all clusters. (F) UMAP projection of all neurons. Color indicates cluster identity. (G) Clusters identified in (F) are mapped onto the position of each cell in the Hb for two biological replicates. Scale bar: 100 μm. (H) Quadruple *in situ* hybridization of Tyrosine Hydroxylase (*Th*), Tachykinin1 (*Tac1*), Choline Acetyltransferase (*Chat*), and Calbindin1 (*Calb1*) mRNA. Scale bar: 100 μm. (I) Quantification of overlap in (H). Grayscale indicates the proportion of cells expressing Gene 1 that also express Gene 2. Fractional overlap listed inside each box. n = 3639 neurons. (J) Left, coronal sections from mouse atlas showing the axonal projections from the medial Hb to the interpeduncular nucleus (IPN) (Konsman, 2001). Top right, neurons expressing AAV1-DIO-EYFP in X-Cre animals in the Hb, with α-GFP immunostaining. Bottom right, 3D rendering of YFP^+^ IPN axons of X-Cre:DIOYFP animals after tissue clearing (~3-mm-thick sections, pseudocolored for YFP intensity). Scale bars: 100 μm. See also Figure S1.

We simultaneously assessed expression and spatial distribution of 15 genes (including neuromodulator receptors and neuropeptide-encoding genes) via tissue sequencing enabled by hydrogel-tissue chemistry (STARmap; Wang et al., 2018 Figures 1A–1C). Consistent with previous reports (Contestabile and Fonnum, 1983; Wagner et al., 2016), these data highlight the non-overlapping expression of *Chat* and *Tac1* populations (Figures 1D and S1A). We identified six transcriptionally defined clusters (Figures 1E and 1F), which occupied different spatial subregions in the MHb (Figures 1G and S1B), in line with recent observations (Hashikawa et al., 2020). One cluster (Hb1) mapped onto dorsal MHb cells that expressed *Tac1* at high levels. Two clusters (Hb2, Hb3) were enriched for *Chat*. Another cluster, Hb4, was enriched for *Th, Calb1, Cartpt*, and *Htr5B*, with cells spatially intermingled in the stria medullaris and Region X (HbX), a suggested region between the MHb and LHb (Seigneur et al., 2018; Wagner et al., 2016; Wallace et al., 2020). Cluster Hb5 was defined by high *Gpr151* expression and the remaining cluster (Hb6) by *Gad2*^+^ neurons located in the LHb (Figure 1G), as described previously (Quina et al., 2020; Wallace et al., 2020).

With the goal of linking molecular markers to functional features, we chose four candidate genes from the STARmap analysis (*Tac1, Chat, Th,* and *Calb1*), as these genes largely defined transcriptionally and spatially distinct neural populations, including dorsal MHb, ventral MHb, and HbX. Expression patterns were confirmed by quadruple *in situ* hybridization (Figures 1H and 1I and S1C and S1D), and injecting a virus driving Cre-dependent expression of yellow fluorescent protein (AAV-Ef1α-DIO-YFP) into transgenic Cre driver lines resulted in YFP expression consistent with the *in situ* data (Figure 1J, top row). Volumetric imaging of the YFP^+^ axon termination fields in the IPN revealed cell type-specific innervation patterns. Tac1^+^ axons terminated laterally, ChAT^+^ axons crisscrossed the midline (Video S1) (Ables et al., 2017; Hsu et al., 2013), and both TH^+^ and Calb1^+^ axons localized to the ventral IPN as a thin sheet (Figure 1J). Taken together, our STARmap sequencing screen identified genetically defined populations with distinct transcriptional, spatial, and axonal-projection properties.

### Cell-type-specific neural dynamics in a visuospatial task

**Figure 2.**
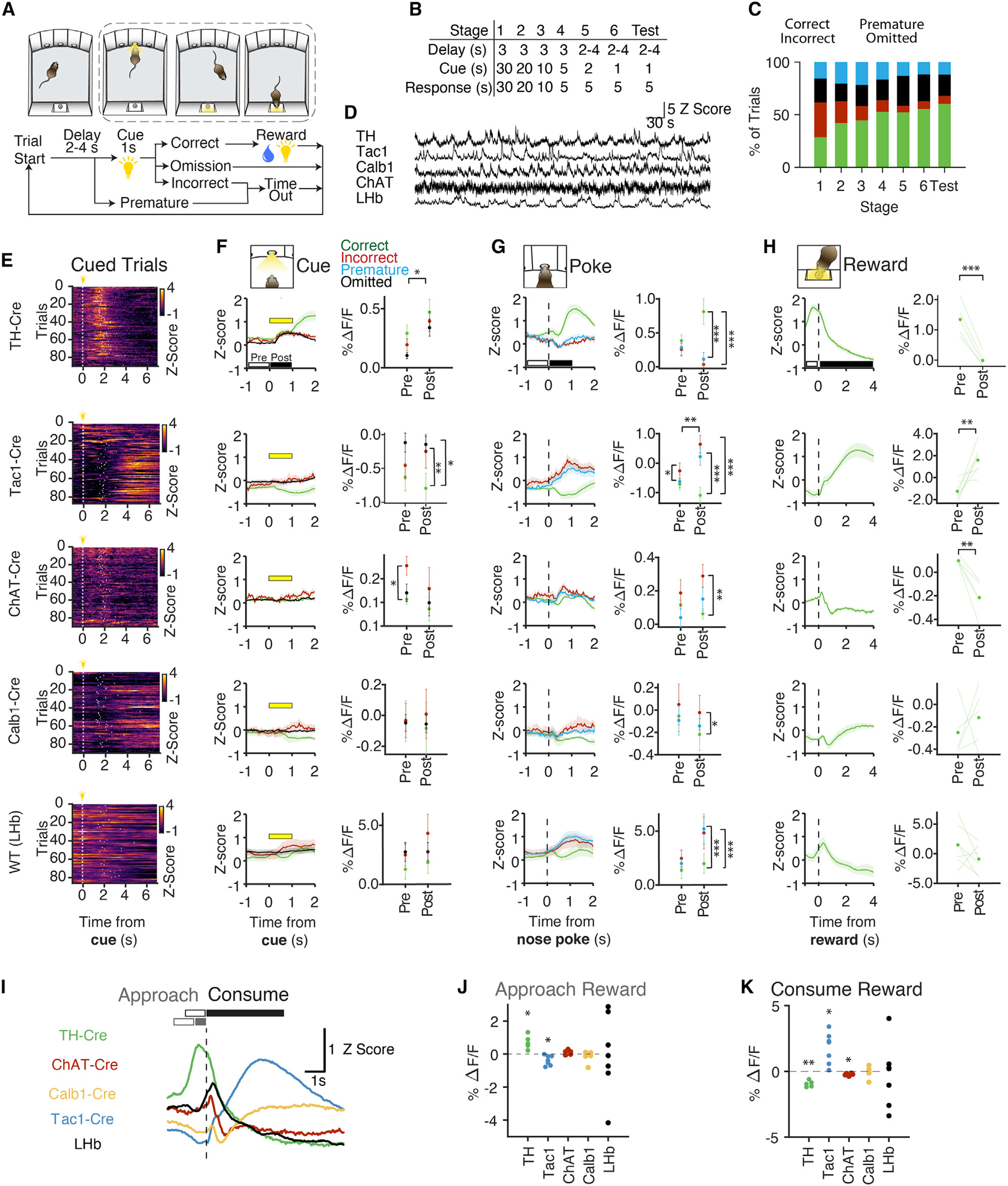
Habenular cell types show distinct reward-related activity. (A) Trial structure of 3-Choice Serial Reaction Time Task. After a variable delay, a cue light appears in one of three nose pokes for 1 s. Nose pokes into the lit port result in delivery of sucrose water at the reward port on the opposite wall. Premature and incorrect trials result in a 5 s time out. (B) Training consists of 6 stages of progressively shorter cue durations and the introduction of a variable delay. (C) Behavioral performance across training. Percentage of correct (green), incorrect (red), omitted (black), and premature responses (blue) for all animals (n = 29 mice). (D) Example traces from photometry recording during behavior, Z scored across each session. (E) Example photometry recordings from one behavioral session per genotype. Each row represents a single trial where t = 0 is cue onset. Color: *Z* scored fluorescence. Data from premature and omitted trials are not displayed. (F–H) Left panels, photometry time series normalized to 405 nm control, Z scored across each session, and aligned to cue onset, nose poke, or reward port entry. Data is separated by behavioral outcome: correct (green), incorrect (red), omitted (black), and premature (blue). Error bars indicate SEM. Right panels, %ΔF/F calculated before and after the cue (F) or nose poke (G). Two-way ANOVA with repeated measures correction. Cue effect: Th^+^, p < 0.05. Nose poke effect: Tac1^+^, p <0 .01. Trial outcome vs cue: Tac1^+^, p < 0.05. Trial outcome versus nose poke: Th^+^, p <0 .01; Tac1^+^, p <0 .001; ChAT^+^, p < 0.05. See Table S1 for multiple comparisons. For reward (H), Th^+^, p <0 .001, Tac1^+^, p <0 .01, ChAT^+^, p <0 .01 by paired t test. (I) Summary of mean *Z* scores for each genotype in (H), aligned to reward port entry. (J and K) Quantification of the change in GCaMP fluorescence at reward approach or reward consumption with a one-sample t test with FDR correction. For reward approach: Th^+^, p < 0.05 and Tac1^+^, p < 0.05. For reward consumption, Th^+^, p < 0.01, Tac1^+^, p < 0.05, ChAT^+^, p < 0.05. In all panels, black bars indicate the time periods analyzed before (open) and after (filled) behavioral events. Data represents average across mice: Th-Cre, n = 5 mice; Tac1-Cre, n = 7 mice, ChAT-Cre, n = 5 mice, Calb1-Cre, n = 5 animals; LHb, n = 7 animals. Error bars: SEM. See also Figure S2 and Table S1.

To directly compare the role of these cell types in behavior, we recorded population activity using fiber photometry from each MHb cell type and one pan-neuronal LHb population in the three-port serial reaction time task (3CSRTT), an operant task that involves learning, visuospatial attention, behavioral inhibition, motivation, and reward expectation (Figures 2A–2C and S2A–S2C) (Bari et al., 2008; Kim et al., 2016). Briefly, animals learned to nose poke to a brief visual cue in one of three ports; upon a correct response, a reward light was illuminated on the opposite wall with 4% sucrose in water delivered below. As mice progressed in training, they developed an attentive scanning behavior (Video S2) and premature pokes shifted to the end of the delay period, indicating cue light anticipation (Figure S2D). We injected AAV1-Ef1α-DIO-GCaMP6f into the MHb of Cre-expressing transgenic mice, or AAVdj-hSyn-GCaMP6m into the LHb of non-transgenic C57BL/6 mice, and recorded photometry signals during the 3CSRTT (Figures 2D–2K and S2E and S2F). Trial-aligned photometry showed distinctly structured activity patterns (Figure 2E). To determine if particular cell types showed activity dynamics correlated with attentional state (cue), behavioral inhibition (nose poke), or rewarded outcomes (reward port entry), we aligned photometry signals to each behavioral epoch and separated neural traces by trial outcome (Figures 2F–2H).

The 3CSRTT requires the animal to attend to the cue panel in anticipation of the cue light, allowing this paradigm to be used to assess visuospatial attention (Robbins, 2002). Only ChAT neurons showed pre-cue activity predictive of trial outcome, suggesting a possible role in the attentional component of the task (Figure 2F). Cue illumination significantly increased activity in TH^+^ neurons for all trial types, indicating that TH neurons may encode salient cues in the environment. We next aligned the data to nose pokes to compare activity before pokes on different trial outcomes (premature pokes are a suggested metric for impulsivity and are altered in MHb-lesioned mice (Kobayashi et al., 2013; Sanchez-Roige et al., 2011). We found only a small difference in correct versus incorrect trials for Tac1^+^ neurons (Figure 2G). In contrast, the post-nose poke period revealed trial outcome-dependent activity in TH^+^, Tac1^+^, ChAT^+^, and LHb populations, with prominent differences between rewarded (correct) versus unrewarded (incorrect or premature) trials. When we aligned data to the reward port entry, TH^+^, Tac1^+^, and ChAT^+^ neural signals were all significantly modulated by reward, but with distinct dynamics (Figure 2H). TH^+^ activity peaked as mice approached the reward port and then diminished quickly. Tac1^+^ activity gradually increased during reward consumption whereas ChAT^+^ activity decreased during this time period. Calb1^+^ and LHb populations were not significantly modulated by reward.

Separating reward approach from reward consumption, we found that TH^+^ neurons were active during reward approach, whereas Tac1^+^ neurons were active during reward consumption (Figures 2I–2K). To rule out movement-related signals, we compared these responses to other (unrewarded) head entries into the reward port. Tac1^+^ neurons increased activity during both rewarded and unrewarded head entries, but with different temporal dynamics (Figures S2G and S2H); unrewarded head entries triggered a subsecond-onset/offset signal whereas rewarded head entries elicited slower dynamics (τ_on_ = 0.22 s unrewarded versus 2.29 s rewarded, τ_off_ = 1.32 s unrewarded versus 2.26 s rewarded). In other cell types, there was no activity change to unrewarded entries. For ChAT^+^ signals, there was little modulation in the 3CSRTT overall, but we observed behaviorally correlated activity in the same cohort of animals while in the elevated plus maze, consistent with a role in anxiety (McLaughlin et al., 2017; Figures S2I and S2J). Together, these data revealed diverse functional properties of transcriptionally defined neural populations in the habenula.

### Habenular cell types respond to reward in a learned and expectation-based manner

**Figure 3.**
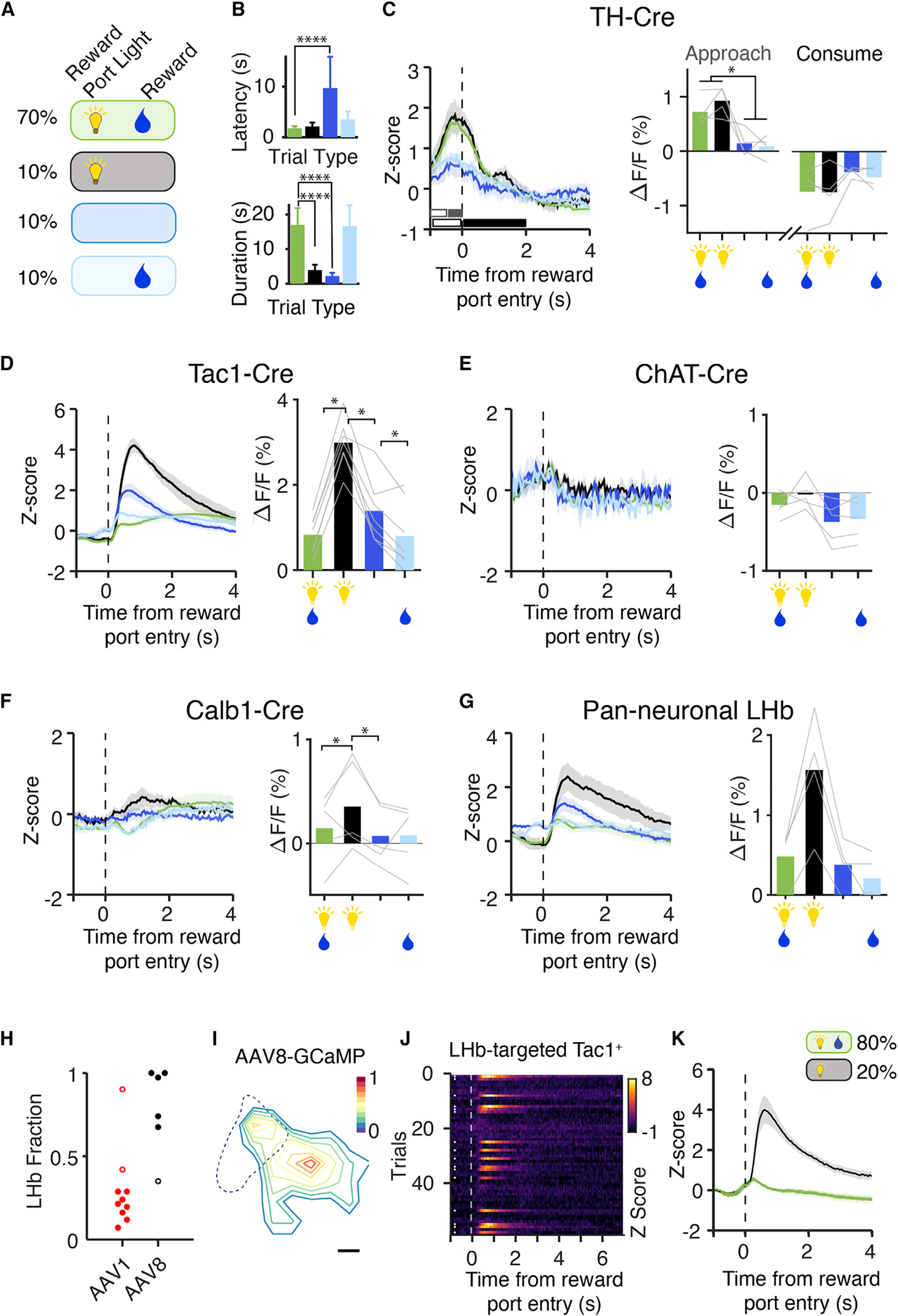
Cell-type-specific segregation of cue-related and outcome-related reward activity. (A) A variant of the task where reward-predicting cues and reward probabilities were modified. In 70% of correct trials, rewards were cued and delivered (green). In the remaining correct trials, the reward was not cued (light blue), the reward was not delivered (black, 10%), or the reward was neither cued nor delivered (navy, 10%). (B) Latency to retrieve reward on correct trials, and the duration in the reward port consuming the reward. Two-way ANOVA with repeated measures, corrected for FRD. For reward latency: cue effect, p < 0.01, reward effect, p < 0.01, Interaction, p < 0.01. For reward delivery: reward effect, p < 0.0001. n = 25 animals. Error bars: SEM. (C–G) Reward-related activity in Hb cell types to predictive cues and reward delivery. Left panels, mean Z scored photometry data aligned to reward port entry. Black and gray bars indicate pre- (open bar) and post- (closed bar) comparison. Color indicates trial type in (A). Right panels, %ΔF/F at reward approach and consumption (C) or consumption (D–G). Gray lines represent individual animals, bars indicate the mean for each reward contingency: TH-Cre, n = 4 mice; Tac1-Cre, n = 7 mice (MHb-Targeted, 82% of neurons in the MHb); ChAT-Cre, n = 5 mice; Calb1-Cre, n = 5 animals; LHb, n = 4 animals. One-way ANOVA with repeated measures and FDR correction, *p < 0.05. (H) Serotype tropism for LHb neurons. The ratio of GCaMP^+^ LHb neurons to all GCaMP^+^ neurons (see STAR Methods) was calculated for AAV1 and AAV8 injections. AAV1, n = 10 animals; AAV8, n = 5 animals. For photometry experiments in Figures 2 and 3, animals with confirmed fiber placement and >70% MHb neurons were included in the analysis in order to assess the activity of MHb Tac1 neurons (the majority of Tac1 neurons in the Hb). Animals excluded from analysis are indicated by open circles. (I) Spatial distribution of LHb-targeted Tac1^+^ neurons. All GCaMP^+^ neurons in the LHb were counted and registered to a common coordinate system. Contour lines: the deciles of normalized cell density. (J) Tac1^+^ neurons in the LHb were targeted for fiber photometry recording using Tac1-Cre mice (88% of neurons in the LHb) and lateral injection of AAV8-DIO-GCaMP6f. Example from one animal in a session where 20% of rewards were withheld, showing all correct trials aligned to the reward port entry. White dots: withheld trials. (K) Mean Z score for LHb-targeted Tac1^+^ neurons at reward port entry for rewarded (green) and withheld (black) trials. n = 7 animals. See also Figure S3.

We next sought to better understand the contingencies of these reward-related signals. We introduced variants of the task in which the reward-predictive cue (reward port light) and reward (sucrose) were unlinked in some trials (Figures 3A–3G), or in which we varied reward size (Figures S3A–S3D). We found that mice entered the reward port more quickly when the port light was on, indicating learned association between the port light and reward. Photometry signals from TH^+^ neurons during the reward period were not significantly different between rewarded and unrewarded trials but were significantly higher when the reward-port light was on (Figure 3C). Video tracking of head angle showed that TH^+^ signals were time-locked to orienting movements toward the reward port (Figures S3E and S3F), but reward-independent changes in luminance (e.g., house light) did not alter TH^+^ activity (Figure S3G). To rule out activity driven by the preceding nose poke, we delivered free sucrose rewards to 3CSRTT-trained mice outside of the trial structure (Figures S3H–S3J). TH^+^ signals increased only when free rewards were cued by a reward port light (Figure S3H), and doubling the reward size had no effect on TH^+^ activity (Figure S3B).

These data suggested that the TH^+^ population can represent a learned reward-predicting cue. To test this hypothesis, we performed a reversal paradigm in trained mice in which the reward-port light was paired with incorrect (unrewarded) trials instead of correct (rewarded) trials. In this paradigm, the cue light was still a salient stimulus, but no longer associated with a reward. We found that the TH^+^ light response decreased across five reversal sessions (Figures S3K–S3M). Together, these experiments strongly suggested that the TH^+^ population can signal a learned encoding of reward-predicting cues. In contrast, in the withheld reward task, Tac1^+^ neurons demonstrated rapid and robust activity on unrewarded trials, particularly those with a reward cue (Figure 3D, black), while ChAT^+^ activity showed no dependence on these reward contingencies, and Calb1^+^ neurons showed only modest differences among trial conditions (Figures 3E and 3F). In summary, we found that TH^+^ and Tac1^+^ neurons signaled distinct task-related features of reward-guided behavior: TH^+^ neurons encoded learned reward-predicting cues and Tac1^+^ dynamics were linked to reward outcome.

### Opposing Tac1^+^ signals resolved by topography

Tac1^+^ photometry signals increased in both rewarded and unrewarded trials. Why would they signal both acquired and missed rewards? The differences in the temporal dynamics of the photometry signal suggested that they might originate from two distinct Tac1^+^ populations. We had targeted our recordings to the MHb, but the LHb also contains Tac1^+^ neurons. Based on previous work, we next hypothesized that LHb Tac1^+^ neurons signaled negative outcomes, whereas MHb Tac1^+^ cells signaled acquired rewards (Figure 3G) (Bromberg-Martin and Hikosaka, 2011; Hsu et al., 2014, 2016; Matsumoto and Hikosaka, 2007).

We implemented a viral tropism targeting strategy to record more specifically from LHb Tac1^+^ neurons (hereafter Tac1^LHb^; Figures 3H, 3I and S2F). We found that Tac1^LHb^ neurons responded reliably to withheld rewards (Figures 3J and 3K), with dynamics matching those in our previous recordings (Figure 3D). Conversely, Tac1^LHb^ recordings did not recapitulate the reward-associated activity seen in the MHb-enriched Tac1^+^ population (Figures 3J and 3K). Together, these data strongly suggested that the opposing valence signals arise from anatomically distinct Tac1^+^ populations.

### Tac1+ MHb cells exhibit long-timescale activity dynamics

**Figure 4.**
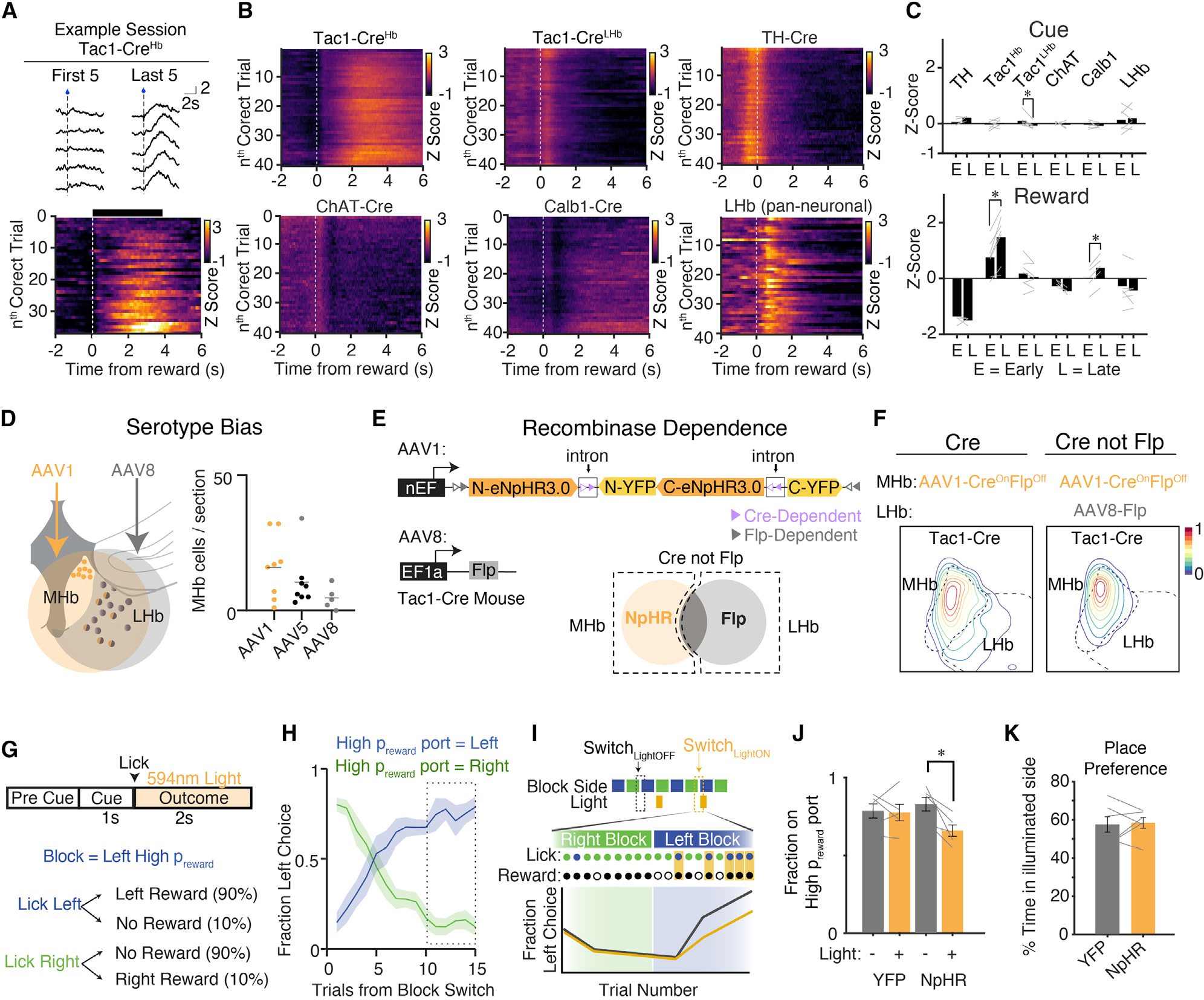
Long-timescale activity dynamics and behavioral significance of habenular cell types. (A) Top, example traces of photometry signal for a Tac1-Cre mouse for the first 5 rewards and last 5 rewards of a behavioral session. Dotted line indicates reward delivery. Bottom, reward responses for correct trials in an example session from one mouse, sorted by n^th^ correct trial. (B) Average reward response for each genotype, *Z* scored across each session and sorted by n^th^ correct trial. Only rewarded trials are displayed. (C) Quantification of fluorescence changes over a behavioral session 1 s after cue onset and 4 s after head entry into the reward port. To look at changes across the session, the mean *Z* score of the first 5 correct trials were compared to 5 late trials (correct trials #36–40). *p < 0.05 by paired t test. Error bars indicate SEM All p values are FDR-corrected. Tac1^Hb^ animals include AAV1 injections with fiber placements in the MHb (82% of GCaMP^+^ neurons in the MHb). Tac1^LHb^ animals include AAV8 injections with fiber placements in the LHb (88% GCaMP^+^ neurons in the MHb). Gray lines: individual animals. (D) Bias for AAV1-YFP, AAV5-YFP, and AAV8-YFP to infect MHb neurons. Injection locations at MHb/LHb boundary were verified with simultaneous fluorosphere injection. Each dot represents one animal. (E) Intersectional strategy to target MHb Tac1^+^ neurons for optogenetic silencing. AAV1 Cre^On^Flp^Off^ eNpHR3.0 injected in the MHb to turn on expression of eNpHR3.0 in all Tac1 Hb neurons. AAV8-Flp preferentially infects LHb neurons to turn off eNpHR3.0 expression laterally. (F) Distribution of virally infected neurons in AAV1 Cre^ON^Flp^Off^ alone versus AAV1 Cre^ON^Flp^Off^ plus AAV8-Flp. Contour lines represent the deciles of normalized cell density. n = 5 animals/condition. (G) Behavioral paradigm for head-fixed reward-guided decision-making task. Animals are presented with two lick spouts. In a block trial structure, one spout hasa high probability of reward (0.9) and the other a low probability of reward (0.1). After a pre-cue period in which premature licks terminate the trial, a 1 s cue light is illuminated. During this time, licks result in delivery of a water reward according to the reward contingencies defined for that block. Optogenetic inhibition is restricted to rewarded trials on the high probability lick spout. (H) Fraction of left side choices on right-to-left block switches (blue) and left-to-right block switches (green). Error bars indicate SEM. Dotted box: trials quantified in (J). (I) Schematic of stimulation paradigm at block switches and prediction of the behavioral response. On a subset of block switches, rewarded licks on trials 0–15 after the block switch trigger 2 s of 594 nm light to activate eNpHR3.0. (J) Mean fraction of high probability port choices for each animal across trials 10–15 after the block switch for control and light inhibited trials (indicated by dotted box in [H]). n = 5 animals. (K) Time spent in yellow light stimulated side of a two-chamber real time place preference assay. n = 6 animals. See also Figure S4.

The trial-averaged data from habenular cell types had revealed interesting differences in reward responses, but we also noticed longer timescale dynamics across behavioral sessions: rewards in early trials triggered little activity in Tac1^+^ neurons, whereas later rewards showed more reliable and robust responses (Figure 4A). In addition, Tac1^+^ reward responses were higher if the previous trial had been rewarded (Figures S4A–S4E), together suggesting a sensitivity to reward history. Tac1^LHb^, TH^+^, and ChAT^+^ populations lacked this ramping activity (Figures 4B and 4C). This pattern was of interest because it suggested that Tac1^MHb^ populations might be important for integrating past reward outcomes and updating behavioral strategies. Testing the causal role of these neurons in reward-guided decision-making required more specific viral targeting of Tac1^MHb^ neurons to yield an interpretable result—particularly because Tac1^MHb^ and Tac1^LHb^ neurons encode opposing valence events. To this end, we developed a method leveraging viral tropism and INTRSECT gene targeting (Fenno et al., 2014, 2020) to express the inhibitory opsin eNpHR3.0 in Tac1^MHb^. We trained Tac1^MHb-eNpHR+^ mice on a head-fixed, reward-guided decision-making task (Figures 4D–4G) in which mice were presented with two lick spouts: one with a high reward probability (90%) and one with a low reward probability (10%). The spout with the high reward port was alternated every 15–20 rewarded trials. Block switches were not signaled; thus, the task required mice to sample both lick spouts and integrate information about recent reward history to optimize reward seeking (Figure 4H).

We delivered precisely timed optogenetic inhibition for 2 s after the first lick on rewarded trials, coinciding with reward-elicited Tac1^MHb^ activity as seen in photometry experiments (Figure 4I). Silencing during this time period degraded adaptive transitions and behavioral performance (Figures 4 and S4F–S4I), though no changes were observed in a real-time place preference assay, suggesting the effect was not due to an aversion related to Tac1^MHb^ inhibition (Figure 4K). Thus, Tac1^MHb^ neuron activity exhibits a functional role in guiding future explore/exploit decisions.

### Individual Tac1^+^ MHb cells ramp up as rewards are gathered

**Figure 5.**
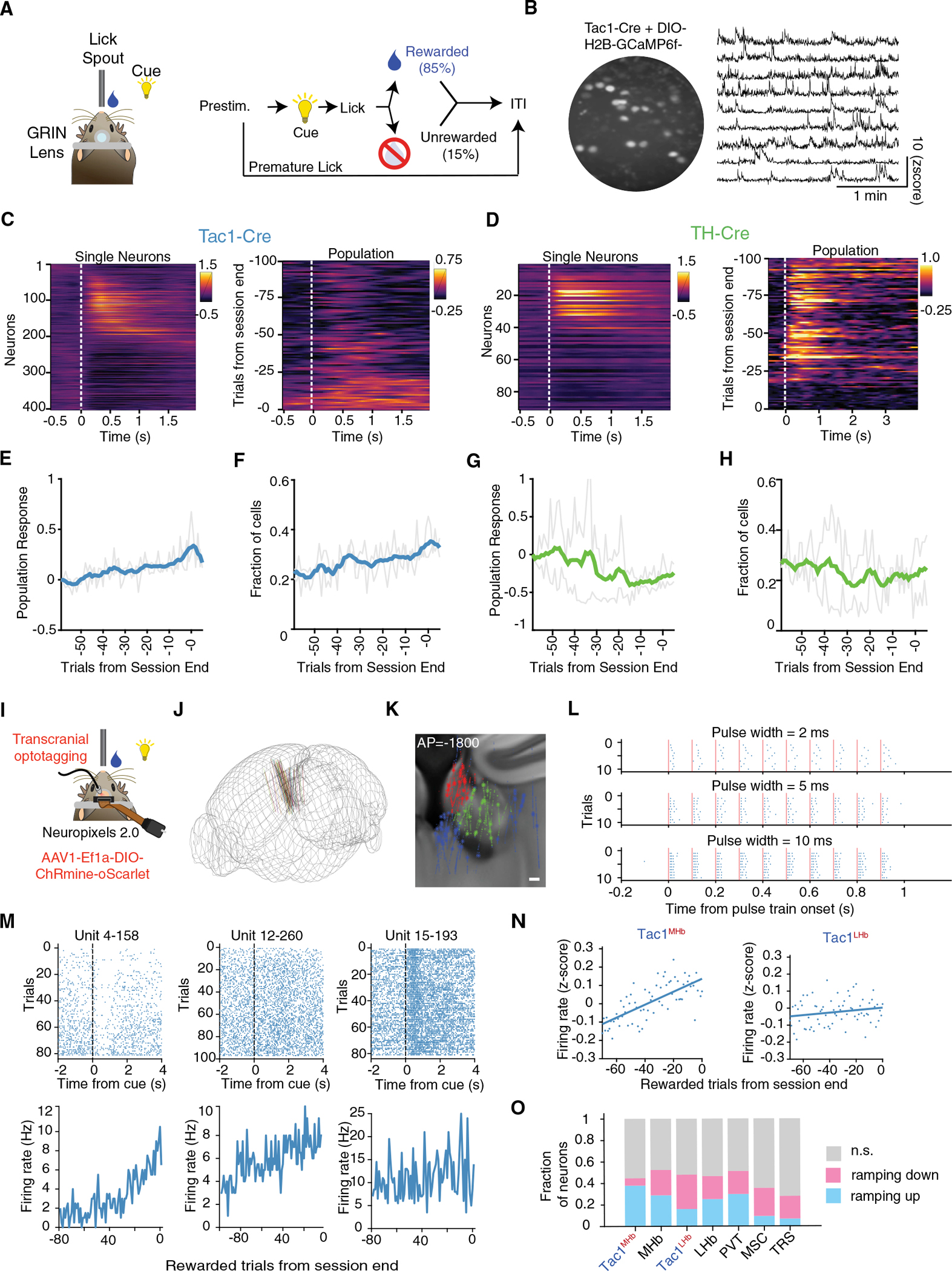
Cell-type-specific imaging and electrophysiology reveals distinct long-timescale dynamics at single-neuron and population level. (A) A simplified reward task for head-fixed calcium imaging and electrophysiology. During a 0.5 s prestimulus period, animals must withhold licking. During a 1 s cue period, licks are rewarded with sucrose water delivery. In 15% of trials, earned rewards are withheld. Trials are separated by a variable ITI. (B) Example 2p image of Tac1^MHb^ neurons expressing H2B-GCaMP6f, imaged through a 600 μm GRIN lens. Right, example traces from Tac1^MHb^ neurons. (C–D) Heatmap showing (left) *Z* scored, trial-averaged activity from single Tac1^MHb^ (C) or TH^+^ (D) neurons on rewarded trials. Neurons from 2 mice are included in each panel. (right) Heat maps from one example animal showing population activity (sum of fluorescence across all neurons) for each trial in one session (right). (E and G) Activity of reward-responsive neurons during the reward period over a behavioral session in Tac1^MHb^ and TH^+^ neurons, respectively. Data normalized to the first trial shown. Mean (bold line) and individual mice (gray lines). Across the session, Tac1^MHb^ populations showed stereotyped and statistically significant increasing population activity (p = 1.7×10^−22^) and TH^+^ populations showed more variable and slightly decreasing population activity (p = 3.6×10^−5^); p values for the null hypothesis that the slope of linear regression is zero. (F and H) The proportion of reward-responsive cells over the session in Tac1^MHb^ and TH^+^ mice, respectively. A responding cell is defined as a cell with a *Z* score greater than 0.25 for the 1 s reward period. Across the session, Tac1^MHb^ neurons show a statistically significant increase (p = 6.4×10^−15^) and TH^+^ neurons show a statistically significant decrease (p = 5.8×10^−4^) in the fraction of active neurons. (I) Experimental configuration for Neuropixels 2.0 recording. A 4-shank probe was approached at 10 from the midline. A 637 nm laser was illuminated above the skull. (J) Summary of Neuropixels probe insertions targeting MHb. n = 7 animals, 18 behavioral sessions. (K) Spatial position of recorded single neurons registered to the Allen Brain Atlas. Red, MHb; green, LHb; blue, others; *, optotagged. See Figure S5C for more complete visualization. n = 6099 Hb neurons, including 29 optotagged Tac1^MHb^ and 25 optotagged Tac1^LHb^. (L) Spike raster plot for an example optotagged Tac1^MHb^ neuron at 2, 5, 10 ms pulsewidth. (M) Spike raster plot and firing rate across trials, for three example optotagged Tac1^MHb^ neurons. (N) Left, population-averaged baseline firing rate across trials for Tac1^MHb^ neurons (p = 4.6 × 10^−16^); right, baseline firing rate across trials for Tac1^LHb^ neurons (p = 0.10). (O) Fraction of neurons in each brain area showing significant ramping up or ramping down across a behavioral session. See STAR Methods for statistical criteria for classifying ramping characteristics. See also Figures S4 and S5.

To investigate these reward signals at single-cell resolution, we used endoscopic two-photon Ca^2+^ imaging of Tac1^+^ or TH^+^ MHb neurons expressing H2B-GCaMP6f. We adapted a simple head-fixed cue-reward association task (Figure 5A), in which a visual cue signaled availability of water reward, which mice obtained by licking a spout (rewarded). In 15% of trials, no reward was delivered (unrewarded). Trial-averaged activity traces revealed that ~40% of Tac1^+^ cells and ~25% of TH^+^ cells were task modulated (Figures 5C and 5D). Averaging the activity of individual neurons revealed cell-type-specific long-timescale activity dynamics as in the photometry recordings (Figures 5C and 4B). In particular, the population-averaged activity of Tac1^+^ (but not TH^+^) neurons displayed the striking ramp-up across trials (Figures 5E–5H), and individual Tac1^+^ neurons also showed clear ramping across trials (Figure S5A).

We complemented these single-cell findings with electrophysiological recordings in the same task. We used the 4-shank Neuropixels 2.0 probes (Steinmetz et al., 2021) and established a reliable workflow to access MHb neurons (Figures 5I–5K). We conferred cell type specificity through deep-brain transcranial optotagging with the opsin ChRmine (Chen et al., 2021; Marshel et al., 2019, Kishi et al., 2022) (Figure 5L). Our recordings spanned the habenular area and the septum, a major input to MHb (Figures S5B–S5E). We again observed ramping in single neurons and populations of the Tac1^MHb^, but not Tac1^LHb^, cell type (Figures 5M and N). All regions had mixtures of ramping-up and ramping-down neurons; however, in the Tac1^MHb^ population, the ramping-up population greatly outnumbered the ramping-down population (Figure 5O).

### Data-driven dynamical systems modeling: Tac1^MHb^ line-attractor dynamics integrate reward history

**Figure 6.**
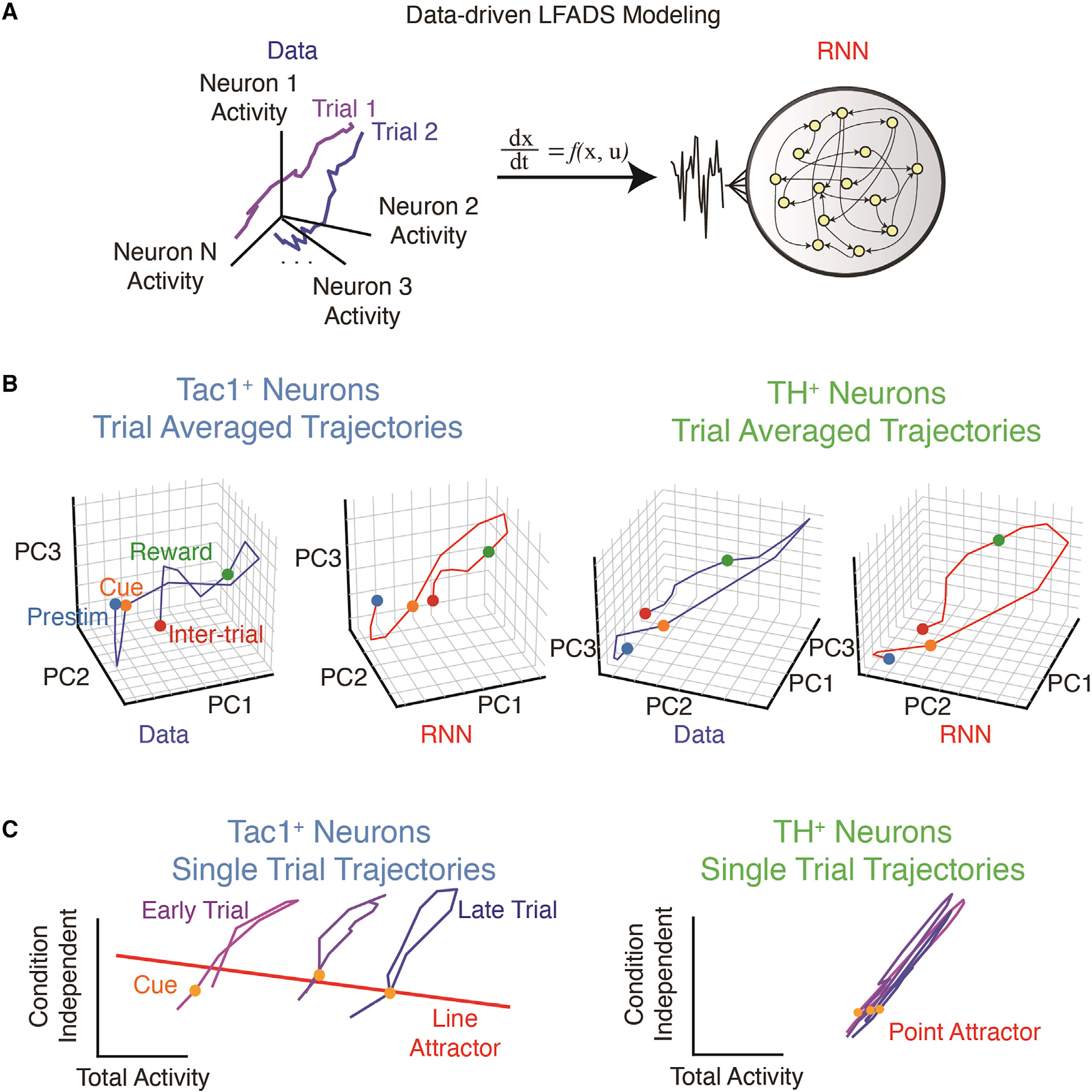
Data-driven modeling of cellular-resolution population activity identifies cell-type-specific attractor dynamics. (A) LFADS modeling of neural population activity. The dynamics of Tac1^MHb^ or TH^+^ neurons measured by two-photon microscopy can be described as trajectories in the neural state space (left). These trajectories can be generated by an RNN, which approximates the underlying neural dynamical system, at the single-trial level (right). (B) The trial-averaged neural trajectory of Tac1^MHb^ (left) and TH^+^ (right) neurons in the raw data and the RNN, demonstrating consistent epoch-dependent dynamics demarcated by the dots. (C) Single-trial trajectories and the underlying attractor manifolds identified by fixed point analysis of the trLFADS generator RNN. In the Tac1^MHb^ neurons (left), the line attractor integrates the external inputs over time, resulting in the trajectories shift along the line attractor as the session progress. Note the alignment of the line attractor and the total activity mode for Tac1^MHb^ neurons, implying the progressive increase of total activity. The straight colored line is the top principal component of the identified fixed points. In the TH^+^ neurons (right), the discrete point attractor confines the trajectories, resulting in no change in total activity over time. The orange dots represent the cue onsets. See also Figure S6.

To leverage and learn from our cellular-resolution and cell-type-specific experimental datasets (Figure 5), we developed a Ca^2+^ imaging-compatible data-driven dynamical systems approach for single trials based on latent factor analysis via dynamical systems (LFADS), a computational technique for inferring single-trial neural population dynamics (Pandarinath et al., 2018a), which we term type-resolved LFADS (trLFADS). Though trLFADS is applicable to both cell-type-specific electrophysiology and Ca^2+^ imaging datasets in principle, here we focus on imaging to harness the largest number of relevant simultaneously recorded neurons, and in so doing, enable LFADS for cell-type-specific activity data for the first time. In essence, trLFADS trains a recurrent neural network (RNN) that regenerates experimentally observed single-trial neural population activity (Figure 6A) by approximating the underlying neural dynamical system f. If x(t) and u(t) represent population state and external input, respectively, where time is t, then trLFADS learns a model $\dot{x}(t)=f(x\left(t\right),\;u(t))$ consistent with the data. The state x(t) is a point in a high-dimensional state space of dimensionality set by the relevant neural latent factors, whose projection corresponds to the activity of individual neurons (Vyas et al., 2020; Pandarinath et al., 2018b). Thus, a single trial can be represented in the neural state space as a trajectory x(t) with time evolution perturbed by the external input (u(t), which is inferred by the model). Here, we modified the network architecture and cost function from previous work (which could only handle spike counts) to enable direct processing of Ca^2+^ transients (STAR Methods). Applying trLFADS to imaging data enabled access both to denoised single-trial neural trajectories and dynamical systems models that generate trajectories.

We trained separate trLFADS models for the separately recorded Tac1^MHb^ and TH^+^ populations. While the trial-averaged trajectories were characterized by a major loop time-locked to cue onset for both Tac1^MHb^ and TH^+^ populations (Figure 6B), denoised single-trial trajectories revealed cell-type-specific structure across the session—a progressive shift across trials in Tac1^MHb^, but not TH^+^, models (Figures 6C and S6A and S6B). Here, population states were shown in two targeted orthogonal dimensions relevant to behavior (which also explained majority of the variance; Figure S6C), with the horizontal axis corresponding to population-averaged total neuronal activity. Within single trials, both Tac1^MHb^ and TH^+^ models revealed a tilted loop trajectory, consistent with the transient increase of total activity after cue onset. Across trials, we found a horizontal procession corresponding to an overall increase in activity, which was specific to the Tac1^MHb^ models (Figure 6C).

To study the dynamical structures governing these trajectories, we next reverse-engineered the learned dynamical systems (generator RNNs in trLFADS) using fixed point analysis (Sussillo and Barak, 2013; STAR Methods). In Tac1^MHb^ models, we identified a continuous line attractor with a large total activity mode projection (Figures 6C and S6A). In contrast, TH^+^ model dynamics were governed by a discrete point attractor (Figures 6C and S6B). Neural attractors represent stable population activation patterns and thus their geometric arrangements provide insights into the logic of the neural dynamics storing information over long timescales. A line attractor integrates external inputs and stores this value as a state, remembering the sum of external stimuli (Mante et al., 2013; Seung, 1996)—as might be expected for the representation of reward accumulation over time.

We next explored the types of external inputs integrated by the Tac1^MHb^ dynamics. We resolved single-trial dynamics into two parts: internal states and external inputs. We quantified condition-averaged inferred external inputs and the corresponding initial state shifts, finding distinct effects between the rewarded and unrewarded trial types (Figures S6D–S6F). Single-trial initial states (i.e., the states before the cue onset) shifted along the line attractor consistent with integration of trial-type-dependent external inputs (i.e., the activity of neural populations upstream to the experimentally observed population)—thus explicitly representing the animal’s reward history, consistent with our findings from the optogenetic reward-guided decision-making experiments (Figure 4).

### Transient optogenetic perturbation and recovery

**Figure 7.**
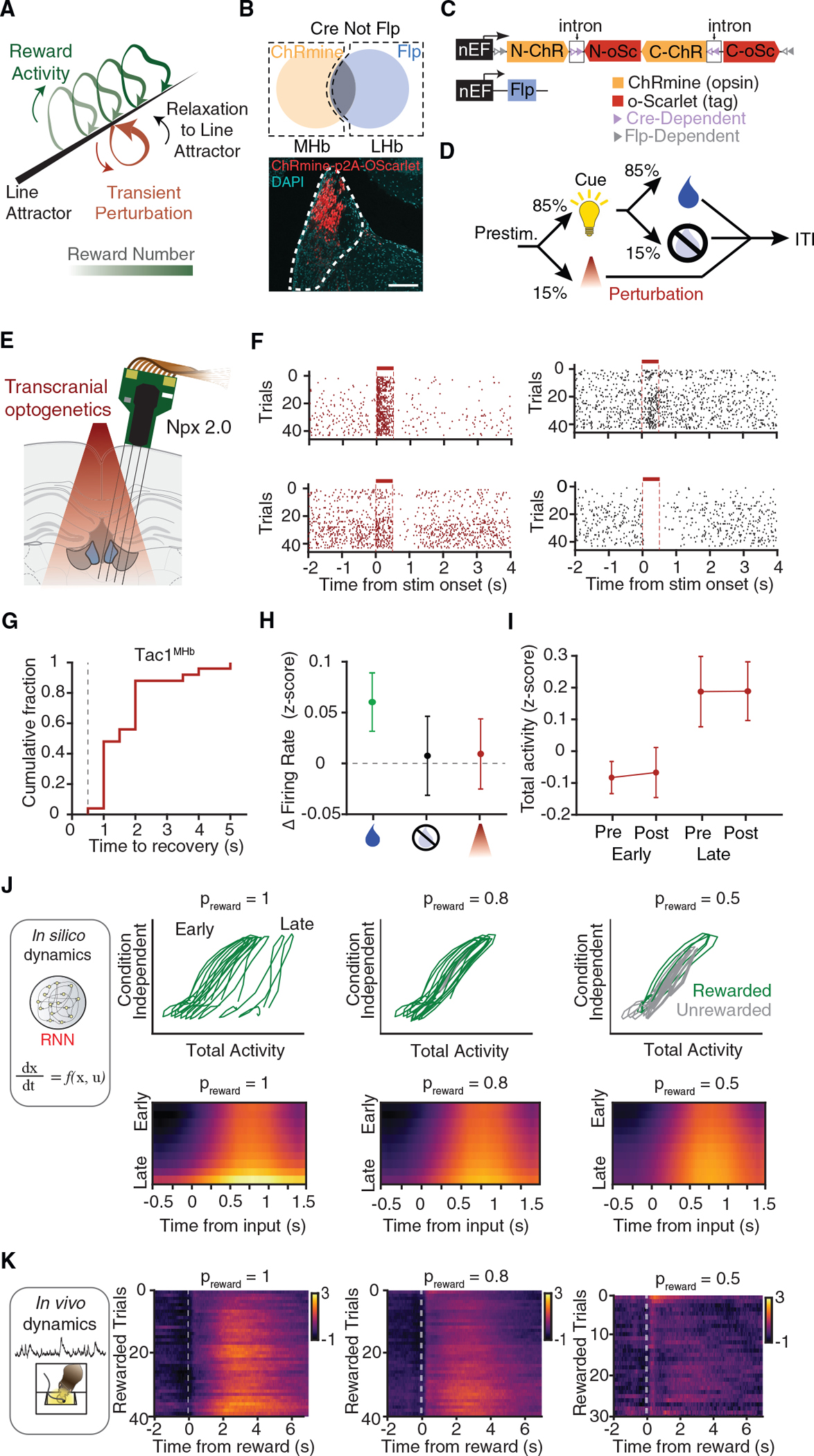
Transient optogenetic perturbation and reward history modulation experiments support the line attractor dynamics model. (A) Schematic representation of transient optogenetic perturbation of the line attractor dynamics. (B) Intersectional gene targeting approach. AAV1 Cre^On^Flp^Off^ ChRmine-p2A-oScarlet is delivered to MHb and LHb neurons of Tac1-Cre mice. AAV8-Flp is delivered to the LHb to turn off ChRmine expression in Tac1^LHb^ neurons. (C) Constructs used for INTRSECT implementation as described in (B). (D) Trial structure for the perturbation experiment. (E) Experimental configuration for transcranial optogenetic stimulation and neural recording using Neuropixels 2.0 probes. (F) Spike raster plots and firing rate changes for example validated optotagged MHb Tac1 neurons (left) or nearby modulated MHb neurons (right), which were all simultaneously recorded. (G) Time to baseline recovery after perturbation for MHb neurons. (H) Average firing rate changes in rewarded (green), unrewarded (black), and perturbation (red) trials. Curves: mean; error bar: SEM from hierarchical bootstrap. n = 1,078 neurons, 4 sessions, 2 mice. (I) Within-trial and across-trial firing rate changes for optogenetic perturbation of MHb Tac1 neurons. Trials were split into early/late halves for concise visualization. (J) Simulation of reward signal accumulation with varying reward probability (1.0, 0.8, and 0.5). Top, example state space trajectories show generated single sessions, initialized at the identical initial state. Bottom, model predictions on changes in total population activity (fiber photometry signal) across rewarded trials. For each case, 1,000 simulated sessions with random initial states were averaged. (K) Fiber photometry recordings in 3CSRTT at 3 different reward probabilities: p_reward_ = 1, p_reward_ = 0.8, and p_reward_ = 0.5. Mean fluorescence (%ΔF/F) during reward as a function of n^th^ correct trial in a session. See also Figure S7.

The identification of line attractor dynamics in the Tac1^MHb^ dynamical systems models generated a series of experimentally testable theoretical predictions. For internal states, we considered that a transiently perturbed neural internal state would be predicted to smoothly and steadily return to the attractor; importantly, this transition would not be instantaneous as it would likely involve multiple cycles of synaptic communication typical of neural attractors. A second prediction was that the projection of external inputs onto the “selection vector” of the line attractor ought to determine the extent of neural state update or integration (Mante et al., 2013); if we pushed the population state in a random direction (nearly orthogonal to a fixed selection vector in high-dimensional neural state space), this transient perturbation would result in nearly zero integration along the line attractor, unlike the well-aligned “reward delivery” input described above (Figure 7A).

Accordingly, we performed brief Tac1^MHb^-specific optogenetic stimulation and electrophysiological recording during the behavior. We used INTRSECT targeting to express ChRmine in Tac1^MHb^ (but not Tac1^LHb^) neurons (Figures 7B and 7C), which enabled transcranial optogenetic perturbation during the head-fixed reward-guided task (Figures 7D and 7E). Transient perturbation robustly triggered diverse types of MHb single-neuron responses (Figure 7F). Optotagged neurons were directly driven by the ChRmine stimulation and thus showed transient excitation. Other modulated units showed both excitation and inhibition, and a substation fraction had seconds-long return-to-baseline dynamics (Figure 7G). These prolonged relaxation kinetics were consistent with network effects predicted by attractor models (Mante et al., 2013).

We included perturbation trials in a range of the baseline states and found that the long-timescale ramping-up dynamics proceeded as expected, independent of the transient perturbations. Because stochastic opsin expression will set an arbitrary multidimensional direction for the transient population-level perturbations (which in this case are not designed to provide naturalistic single-cell-resolution dynamics), guided by the above theoretical predictions we hypothesized that the transient stimulation itself would not contribute to ramping. Quantification of within-trial firing rate changes found that only rewarded trials, but not unrewarded trials or perturbation trials, contributed to the ramping (Figures 7H and S7A and S7B). In summary, consistent with theoretical predictions on transient, unaligned perturbation of line attractor dynamics models, we observed within-trial relaxation onto the pre-stimulation baseline in the Tac1^MHb^ population without significant integration, in contrast to across-trial movement of the baseline by interleaved rewarded trials (Figure 7H).

### Reward history integration *in silico* and *in vivo*

We finally asked if trial-type-dependent external inputs would be sufficient to generate the long-timescale dynamics via cell-type-specific attractors. A crucial feature of data-driven system identification with trLFADS is the possibility for *in silico* neural dynamics experiments, by simulating temporal evolution of neural trajectories across trials while injecting trial-type-dependent external inputs into the learned dynamical systems; the state evolution of these dynamical systems then generates *de novo* neural trajectories (Figure 7J and STAR Methods). We discovered that injecting only rewarded-trial external inputs shifted the neural state along the line attractor, strikingly consistent with our photometry, imaging, and electrophysiology results. In contrast, the same type of simulation with TH^+^ models showed no activity increase over time, consistent instead with operation of a discrete point attractor (Figure S7C).

Finally, we considered how different reward probabilities might modulate these dynamics. Computational titration of reward delivery probability was carried out by varying the ratio of rewarded- and unrewarded-trial external inputs; these *in silico* experiments assessed reward probabilities not yet experimentally tested (p_reward_ = 0.5, 0.8, or 1.0). Differential slopes of activity accumulation were predicted in Tac1^MHb^ but not TH^+^ simulations (Figures 7J and S7C). We compared the simulated dynamics to experimentally measured neural dynamics from photometry signals in freely moving mice. We found that ramping developed more steeply at higher reward-probabilities (Figure 7K), consistent with the trLFADS prediction and supporting the model that Tac1^MHb^ population dynamics can serve as a line attractor that integrates reward history.

In summary, by computationally reverse-engineering the trained trLFADS models to generate testable hypotheses, we predicted dynamics consistent with a cell-type-specific line attractor system for Tac1^MHb^ neurons. This outcome may thus reveal a computationally defined cell type marker (line-attractor integration of reward history), which in this case maps onto a specific genetically defined and regionally localized cell type.

## DISCUSSION

Here, we measured neural population activity from multiple habenular cell types during rewarded behavioral tasks. We began by providing the initial report of temporal structure and probability-context dependence of habenular Tac1^+^ neuron responses and showed TH^+^ cells learn to encode reward-predictive cues (Figures 2 and S3E–S3G) (consistent with previous work suggesting separate roles for cholinergic and peptidergic MHb cell types; Seigneur et al., 2018). Next, through dynamical system modeling we identified a cell-type-specific line attractor (Tac1^+^ but not TH^+^) underlying long-timescale reward history integration, a computation important for establishing the value of actions over many behaviorally relevant timescales, particularly in environments with sparse or dynamic rewards (Hattori et al., 2019; Sugrue et al., 2004; Hwang et al., 2017; Kawai et al., 2015; Morcos and Harvey, 2016; Scott et al., 2017; Sul et al., 2010; Zalocusky et al., 2016; Bayer and Glimcher, 2005; Bromberg-Martin et al., 2010; Lazaridis et al., 2019; Ottenheimer et al., 2018; Tsutsui et al., 2016). These results show how the population activity of a genetically defined cell type can operate as a discrete computational element subserving complex, behaviorally important brain functions.

### TH^+^: Cell-type-specific segregation of predictive valence

Unexpected rewards are known to activate dopaminergic neurons, but after repeated reward exposures, neural activity shifts back in time to cues that precede and predict rewards (Schultz et al., 1997)—a milestone discovery validating prior models of reinforcement learning (Sutton and Barto, 1990). TH, an obligatory enzyme for dopamine synthesis, is often used to mark dopaminergic neurons; however, our TH^+^ neurons are likely “dopaergic” rather than dopaminergic, since they lack other molecular machinery for dopamine processing and release aromatic acid acid decarboxylase (AADC) and vesicular monoamine transporter (VMAT2); Weihe et al., 2006) and may produce little of the TH enzyme itself (Lammel et al., 2015). Here, we find *TH* gene expression marks a group of neurons activated by reward-predicting cues. In the future, it will be interesting to explore how TH^+^ neuron activity in MHb/HbX develops before and during training—and perhaps is modulated by how reliably the cue predicts reward availability—akin to neurons in the LHb encoding the information content of a stimulus (Bromberg-Martin and Hikosaka, 2011).

### Habenular pathways of reward and aversion

The habenula has been described as an anti-reward center, though this characterization has been predominantly informed by the activity of LHb neurons because of their response to aversive stimuli and negative reward prediction error (Hikosaka, 2010; Proulx et al., 2014) and to a lesser extent the ventral MHb ChAT^+^ neurons, which have been implicated in anxiety (Cho et al., 2019; Mathuru et al., 2013; Seigneur et al., 2018; Yamaguchi et al., 2013) and aversion (Buolos et al., 2020; Frahm et al., 2011; McLaughlin et al., 2017; Morton et al., 2018; Choi et al., 2021). Evidence of a parallel *positive* reward pathway in Hb had been suggested by observations that Hb lesions can block intracranial self-stimulation (Morissette and Boye, 2008), and more recently by dorsal MHb (predominantly Tac1^+^) optogenetic manipulations demonstrating a role in primary reinforcement and hedonic value (Hsu et al., 2014, 2016). Our data show opposing valence signals for MHb and LHb Tac1^+^ neurons and provide additional evidence of parallel, anatomically distinct reward and anti-reward pathways in the habenula.

The integration of reward history is a computation important for establishing value of actions over a range of behaviorally relevant timescales, particularly in environments with sparse or dynamic rewards. Recent-history effects have been observed in several rewarded tasks and species (Hattori et al., 2019; Sugrue et al., 2004; Hwang et al., 2017; Kawai et al., 2015; Morcos and Harvey, 2016; Scott et al., 2017; Sul et al., 2010; Zalocusky et al., 2016; Bayer and Glimcher, 2005; Tsutsui et al., 2016), including in the LHb (Lazaridis et al., 2019; Bromberg-Martin et al., 2010); further work is needed to test how reward history computations in upstream brain regions might influence Tac1^MHb^ activity. Moreover, the functional impact of Tac1^MHb^ ramping on *downstream* targets remains an open question. The gene Tac1 encodes preprotachykinin, the peptide precursor of Neurokinin A and Substance P; the latter is typically associated with pain and itch (Davidson and Giesler, 2010), but more recent work has shed light on the role of these peptidergic neurons in encoding hedonic value (Hsu et al., 2014) and novelty (Molas et al., 2017). Substance P is released from dense core vesicles upon high frequency stimulation, and it will be interesting to determine if ramping activity may be sufficient to push Tac1^+^ neurons into a peptidergic regime, which has been shown to promote plasticity at MHb-IPN synapses (Melani et al., 2019).

### Cell-type-specific attractor dynamics revealed by data-driven modeling

While it is not trivial for short single-neuron time constants to specifically influence persistent population activity on timescales relevant for adaptive behavioral changes, attractor dynamics implemented in neural systems could in theory serve this bridging role and thus implement aspects of phenomena such as cognitive integration (Robinson, 1989; Seung, 1996), foraging (Hattori et al., 2019), and associative memory (Hopfield, 1982). Experimental observations have identified putative attractor dynamics in certain brain circuits (Finkelstein et al., 2021; Inagaki et al., 2019; Kim et al., 2017; Mante et al., 2013; Miri et al., 2011); however, previous work has not implicated specific cell types in attractor implementation, nor provided direct correspondence to single-trial data crucial for determining if the attractor dynamics could guide individual choices made by behaving animals. Here, we have carried out data-driven identification of the dynamical systems capable of regenerating the relevant experimental neural data (Pandarinath et al., 2018b; Yu et al., 2009; Zhao and Park, 2017; Zoltowski et al., 2020) and directly mapped the underlying attractor dynamics in the resulting models. We were able to quantitatively define cell-type-specific single-trial attractor dynamics, successfully test theoretical predictions describing neural attractor dynamics responding to perturbation, and use *in silico* experiments to simulatevarying reward probabilities.

Many opportunities for exploration remain, especially in the use of optogenetics to further probe these long-timescale flexible changes in behavior. Although we observed nearly zero integration along the putative Tac1^MHb^ line attractor upon recovery from transient one-photon optogenetic perturbation (a perturbation that by design does not match naturally occurring ensemble dynamics), we anticipate that *in vivo* cellular-resolution two-photon optogenetic perturbation (Prakash et al., 2012) of multiple individually defined single cells during behavior (Jennings et al., 2019; Marshel et al., 2019; Carrillo-Reid et al., 2019) precisely aligned to the putative line attractor’s selection vector might be sufficient to result in nonzero integration, further advancing population activity level cellular-resolution causal neuroscience. Such ensemble-optogenetics intervention may be designed to elicit effects on the system that are either matched or mismatched to effects of natural inputs (e.g., from upstream structures such as septum). These future experiments may provide insight into the robustness and uniqueness of the putative line attractor (e.g., testing the possibility that MHb activity may represent readout of upstream populations implementing the identified computations). In addition, although here we studied a single cell type at a time (equivalent to observing the neural states projected onto a specific neural subspace), simultaneous access to multiple cell types may provide insight into higher-order cell type markers and the computations arising from interactions between cell types.

While we and others have previously described the brain-spanning activity arising from even simple behaviors, using electrophysiological recordings without cell type specificity (Allen et al., 2019; Steinmetz et al., 2019), our current results reveal logic linking specific neural population dynamics (often implicated in higher-level computation) to discrete cell types. Identifying specific cell types involved in integration of reward history is important for understanding the design, adaptation, and function of value computation over behaviorally relevant timescales (Roy et al., 2021). Laying groundwork for this direction of investigation, we show how dynamical systems modeling of specific cell types generates testable predictions in the form of computational cell type descriptors; this approach may be broadly useful in elucidating how principles of nervous system function arise from properties of constituent cellular elements.

### Limitations of the study

A common assumption for dynamical systems modeling is that the neural circuit is “fixed” during the behavioral session. This invariance in time is the basis of widely used trial averaging and provides the inductive bias to identify underlying dynamical systems. The fixed-circuit assumption has historically been justified by the fact that animals are typically heavily trained to tasks before neural recordings, with stable performance metrics attained (training was also completed in the present study). However, structural changes due to plasticity may still occur, which may have multiple timescales and mechanisms. For example, synaptic potentiation or cell-autonomous excitability changes might also be able to implement reward history computation, by Tac1^MHb^ or other populations, in the absence of causally relevant line attractor dynamics. A promising approach to test this possibility would involve all-optical electrophysiology (Fan et al., 2020) using voltage sensors for direct synaptic strength testing and excitability measurements *in vivo*. Although application of these emerging optical approaches to deep brain regions such as the habenula will be technically challenging, this approach will be useful even beyond plasticity studies (for example, by revealing mechanistic details of biological substrates underlying the line attractor dynamics, such as local versus multi-regional synaptic contributions).

Our analyses rely on single genes (or a combination of anatomical and genetic properties) to access cell types, but further molecular refinement will be possible or even likely. Our markers captured differences in activity of habenular neurons, but some cell types showed mixed responses (Calb1^+^), and our cellular resolution imaging indicated that not all monogenetically defined neurons exhibit specific reward- or cue-related responses. Recent transcriptional analyses in the habenula have noted that individual genes may be insufficient to unambiguously identify transcriptional cell type (Hashikawa et al., 2020; Wallace et al., 2020; Pandey et al., 2018), and intersectional viral targeting methods (Fenno et al., 2014, 2020) may further refine transcriptome-to-function mapping.

## STAR★METHODS

### RESOURCE AVAILABILITY

#### Lead contact

Further information and requests for resources and reagents should be directed to and will be fulfilled by the lead contact, Karl Deisseroth (deissero@stanford.edu).

#### Materials availability

All unique/stable reagents generated in this study are freely available from the lead contact with a completed Materials Transfer Agreement.

#### Data and code availability

- All code is available on GitHub. Modeling code is available at https://github.com/google-research/computation-thru-dynamics. Photometry code is available on https://github.com/SylwestrakLab/HbRewardCellTypes. *In vivo* electrophysiology code is available on https://github.com/youngju-jo. STARmap data is available on the NeMO Archive (NeMO: 9ACQ8G1, https://assets.nemoarchive.org/dat-9ACQ8G2) and electrophysiology data is availbe on the DANDI archive (DANDI: 000302, https://dandiarchive.org/dandiset/000302).
- Any additional information required to reanalyze the data reported in this paper is available from the lead contact (KD) upon request.

### EXPERIMENTAL MODEL AND SUBJECT DETAILS

Animal husbandry and all aspects of animal care and euthanasia as described were in accordance with guidelines from the NIH and have been approved by members of the Stanford Institutional Animal Care and Use Committee and University of Oregon Institutional Animal Care and Use Committee. For *in situ* sequencing, *in situ* hybridization, and serotype testing, C57/Bl6 mice aged 10w-16w were obtained from Jackson Laboratories (#000664). This study includes data from both male and female mice. For transgenic experiments Tac1-Cre (B_6_; 129S-*Tac1^tm1.1(cre)Hze^*; Jax #021877), Th-Cre (Th^thm1(cre)Te^; MGI:3056580), ChAT-Cre (Tg(Chat-cre) GM24Gsat/Mmucd; MMRRC #017269-UCD), and Calb1-Cre (B_6_.Cg-*Calb1^tm1.1(folA/cre)Hze^*/J; Jax #023531) mice were used.

### METHOD DETAILS

#### STARmap *in situ* sequencing

The STARmap experiment was conducted as previously described (Wang et al., 2018). In brief, glass-bottom 12-well plates were treated by methacryloxypropyltrimethoxysilane (Bind-Silane) and poly-L-lysine (Sigma) following manufacturer’s instructions. Freshly harvested mouse brains were immediately embedded in O.C.T., snap-frozen by liquid nitrogen, and cut on a cryostat to 16-μmm slices. Slices containing habenula regions were mounted in the pretreated glass-bottom plates. Brain slices were fixed with 4% PFA in PBS at 22°C for 10 min, permeabilized with −20°C methanol, and then placed at −80°C for 15 min before hybridization. SNAIL probes were dissolved at 100 mM in ultrapure RNase-free water and pooled. The samples were taken from −80°C and equilibrated to r.t. for 5 min, washed by PBSTR (0.1% Tween 20, 0.1 U/μL SUPERase·In in PBS) for 2–5 min and incubated in 1 × hybridization buffer (2X SSC, 10% formamide, 1% Tween 20, 20 mM RVC, 0.1 mg/mL salmon sperm DNA and pooled SNAIL probes at 100 nM per oligo) in 40°C humidified oven with gentle shaking overnight. The samples were then washed for 20 min twice with PBSTR, followed by one 20 min wash in 4X SSC dissolved in PBSTR at 37°C.Finally, the sample was briefly rinsed with PBSTR once atr.t. The samples were then incubated for 2 h with T4 DNA ligation mixture (1:50 dilution of T4 DNA ligase supplemented with 1X BSA and 0.2 U/μL of SUPERase-In) at room temperature with gentle agitation. Then samples were washed twice with PBSTR, incubated with RCA mixture (1:50 dilution of Phi29 DNA polymerase, 250 mM dNTP, 1X BSA and and 20 μM 5-(3-aminoallyl)-dUTP) at 30°C for 2 h under agitation. The samples were next washed twice in PBST (PBSTR omitting SUPERase·In) and treated with 20 mM Acrylic acid NHS ester in PBST for 2 h at r.t. The samples were briefly washed with PBST once, then incubated with monomer buffer (4% acrylamide, 0.2% bis-acrylamide, 2X SSC) for 30 min at RT. The buffer was aspirated and 10 μL of polymerization mixture (0.2% ammonium persulfate, 0.2% tetramethylethylenediamine dissolved in monomer buffer) was added to the center of the sample, which was immediately covered by Gel Slick coated coverslip and incubated for 1 h at r.t., then washed by PBST twice for 5 min each. The tissue-gel hybrids were digested with Proteinase K mixture (0.2 mg/mL Proteinase K, 20 mM Tris = 7.5, 100 nM NaCl, 1%SDS) at 37°C for 2 h, then washed with PBST three times (5 min each). For *in situ* sequencing, each sequencing cycle began with treating the sample with stripping buffer (60% formamide, 0.1% Triton X-100) at r.t. for 10 min twice, followed by three PBST washes, 5 min each, then sequencing ligation mixture (Wang et al., 2018). Images were acquired using Leica TCS SP8 confocal microscopy with a white light laser, 40× oil-immersed objective (NA 1.3), with a voxel size of 78 × 78 × 315 nm.

#### In situ hybridization

##### Procedure

To prepare tissue for *in situ* hybridization, C57/Bl6 mice aged 10–12 weeks were anesthetized with isoflurane and rapidly decapitated. Brain tissue was immediately removed, embedded in OCT and flash frozen in liquid nitrogen. Tissue was equilibrated to −20°C and sectioned on a microtome (Leica CM500) to a thickness of 20μm and mounted on superfrost plus slides (Fisher Scientific). Tissue was promptly fixed in 4% PFA for 10 min, then transfer to prechilled methanol (−20°C) and incubated at −80°C for 1 h. Tissue was washed two times in 5xSSC, then incubated in hybridization buffer for 30 min. Probes targeting *Tachykinin1*, *Tyrosine hydroxylase*, *Choline Acetyltransferase*, and *Calbindin1* mRNA were designed by Molecular Technologies and diluted to 2nM final concentration in hybridization buffer. Hybridization buffer with probes was added to the slides, covered with Hybrislips, and incubated overnight in a humidified chamber at 37°C. Using a Coplan jar, two washes of 30% formamide in 5xSSC were performed, followed by 2 washes in 5xSSCT. Slides were equilibrated in amplification buffer for 30 min. During this time, fluorophore-labeled hairpins were heated to 95°C for 90 s, then cooled to room temperature at bench top for 30 min. Cooled hairpins were added to amplification buffer and the resulting solution was added to tissue sections and coverslipped. Amplification reaction was run overnight at room temperature, protected from light exposure. Amplified sections were washed 4 times with 5xSSCT in a Coplan jar and then coverslipped for confocal imaging.

**Table:** 

30% probe hybridization buffer	For 40 mL of solution

30% formamide	12 mL formamide
5× sodium chloride sodium citrate (SSC)	10 mL of 20× SSC
9 mM citric acid (pH 6.0)	360 μL 1 M citric acid, pH 6.0
0.1% Tween 20	400 μL of 10% Tween 20
50 μg/mL heparin	200 μL of 10 mg/mL heparin
1× Denhardt’s solution	800 mL of 50× Denhardt’s solution
10% dextran sulfate	8 mL of 50% dextran sulfate

**Table:** 

30% probe wash buffer	For 40 mL of solution

	Fill up to 40 mL with ultrapure H2O
30% formamide	12 mL formamide
5× sodium chloride sodium citrate (SSC)	10 mL of 20× SSC
9 mM citric acid (pH 6.0)	360 μL 1 M citric acid, pH 6.0
0.1% Tween 20	400 μL of 10% Tween 20
50 μg/mL heparin	200 μL of 10 mg/mL heparin

**Table:** 

Amplification buffer	For 40 mL of solution

	Fill up to 40 mL with ultrapure H2O
5× sodium chloride sodium citrate (SSC)	10 mL of 20× SSC
0.1% Tween 20	400 μL of 10% Tween 20
10% dextran sulfate	8 mL of 50% dextran sulfate

**Table:** 

5×SSCT	For 40 mL of solution

	Fill up to 40 mL with ultrapure H2O
5× sodium chloride sodium citrate (SSC)	10 mL of 20× SSC
0.1% Tween 20	400 μL of 10% Tween 20
	Fill up to 40 mL with ultrapure H2O

#### Viral injections

Mice were anesthetized with isoflurane and stereotaxic injections were used to deliver 200–300nL of virus to the medial habenula (AP: −1.4mm, ML +/− 0.3mm, DV, −3.05mm) at a rate of 10 nL/min. For serotype quantification experiments, fluorescent beads (Lumafluor, diluted 1:10) were added to determine center of injection location to verify targeting to the MHb/LHb boundary. Viruses used in the study were generated at the Stanford Viral Vector Core and are listed in the Key Resources Table

#### TMP injections

For Calb1-Cre animals, Cre was under the control of trimethroprim (TMP). To induce Cre, animals were given an i.p. injection of TMP (15 mg/kg). To prepare TMP, a stock solution of 100 mg/ml in DMSO was diluted 1:10 in sterile saline and immediately injected into the animal. All TMP injections were made at least one week after surgery.

#### Tissue clearing

For hydrogel-based cleared tissue experiments, Tac1-Cre, TH-Cre, Calb1-Cre, or ChAT-Cre animals were injected with AAV1-DIO-YFP virus, allowed to recover for 3 weeks, deeply anesthetized with Beuthanasia, and transcardially perfused with 1% hydrogel solution. Brain tissue was removed and post fixed in hydrogel solution overnight at 4C. Dissolved oxygen was removed under vacuum and samples were incubated at 37C for 5 h in degassed hydrogel solution. Tissue was transferred to clearing solution and incubated at 37°C until clear (approx. 2–3 weeks). Clearing solution was refreshed daily for the first week, then every other day until clear. For refractive index matching, tissue was transferred to 25% glycerol overnight, then 50% glycerol for 1–3 h, followed by 65% glycerol until transparent (approx. 2 h). Images were acquired on a Leica SP5 Confocal microscope using a 10× objective. Image planes were acquired at 5 μm intervals. Three dimensional renderings were generated by Imaris Software.

#### CLARITY hydrogel solution

**Table:** 

Stock Solution	Volume	Final Concentration

Acrylamide (40%)	10 mL	1%
Bis (2%)	2.5 mL	0.00625%
VA-044 Initiator	1g	0.25%
10X PBS	40 mL	1×
16% PFA	100 mL	4%
H_2_O	247.5 mL	–

#### SDS clearing solution

**Table:** 

Stock Solution	Volume	Final Concentration

Borate Buffer (5M)	1 L	1M
SDS (20%)	1 L	4%
H_2_O	3 L	–

#### Animal behavior

In preparation for behavioral testing, animals were transferred to reverse light cycle rooms at least 1 week prior to testing. Animals were handled a minimum of 5 days prior to the onset of behavior training. Mice were acclimated to sucrose either by replacing cage water with a 4% sucrose solution, or by allowing animals already under water restriction temporary access to a 10% sucrose solution until animals consumed their day’s allotment of water. Animals were weighed and water restricted prior to the onset of training and weight was monitored to ensure animals did not lose >20% of body weight. For 3CSRTT training, operant conditioning boxes from Med Associates were fit with a custom 3D printed reward wall so as to minimize the contact between the fiber optic emanating from the animal’s skull and the wall of the chamber when then animal was consuming the water reward. The standard Med Associates Five-Choice Serial Reaction Task was modified to include a mid-box beam break to start trials since animals consumed the water reward for a variable duration. In addition to the measurements acquired in the standard protocol, premature poke latency and reward consumption duration were also recorded.

At the onset of training, animals were first acclimated to the operant box for 15 min on Day 1, during which time the house lights were extinguished and the nose pokes were covered. On Day 2, house lights were illuminated and free sucrose water rewards were delivered at variable intervals in the reward port and the reward port light was illuminated until 500 ms after head entry into the reward port. When animals retrieved water rewards with low latency (<5s), nose pokes were uncovered and all three nose poke cues were illuminated. Animals were required to poke into any lit nose poke to retrieve a reward in the reward port (nonspatial training). When a mouse completed 30 trials in 60 min, it was moved onto Stage 1 of the training regimen, from which point onward only one nose poke was active per trial. When animals met the criterion indicated below, they were moved to the next training stage. For most sessions, animals performed 100 trials, or 60 min, whichever came first. In cases with poor performance, additional trials were run to enable animals to reach their daily quota for water to maintain weight. If additional water was required to maintain weight, the remaining water was delivered >1 h after the end of the training session.

#### Stage parameters

**Table:** 

Stage	Cue Duration (s)	Limited Hold (s)	ITI

Stage 1	30	30	3
Stage 2	20	20	3
Stage 3	10	10	3
Stage 4	5	5	3
Stage 5	2	5	2,3,4
Stage 6	1	5	2,3,4

#### Advancement criteria

**Table:** 

Stage	%Correct	% Accurate	% Omitted

Stage 1	30	–	–
Stage 2	40	–	–
Stage 3	50	–	–
Stage 4	50	80	–
Stage 5	50	80	20
Stage 6	50	80	20

#### Elevated plus maze

Animals previously trained on the 3CSRTT were placed in an elevated plus maze, containing 4 arms 35cm in length and 5cm in width. Two arms contained walls 20cm high (closed arms). The entire apparatus was elevated 60cm off the floor. Animals were placed at the center of maze to begin and fiber photometry was recorded for 10 min while the animals explored the maze. Fiber photometry data and video (10 fps) were acquired with a TDT RZ5 using Synapse software.

#### Real time place preference

A custom clear acrylic arena (44 cm × 24 cm × 27cm) was divided into two chambers with unique flooring and wall patterns in each chamber. The arena was recorded from above at 20Hz by a commercially available USB webcam (https://www.elpcctv.com/ELP-USBFHD06H-SFV(2.8-12)). For optogenetic stimulation, mice with fiber optic implants (ThorLabs, CFM32L20) were connected to either a yellow (593nm) or blue (473nm) laser (OptoEngine LLC) via a patch cord (Thorlabs, TT200R5F1B and BFY32FL1). On the day prior to experimentation, mice were acclimated to the arena for 20 min while attached to the fiber optic rotary joint patch cord. The Track-Control toolkit, an open-source object detection and closed loop feedback toolbox run through Python (Zhang et al., 2019) was used to manually assign the stimulated chamber at the start of each experiment, and track the centroid of the mouse. When the centroid of the mouse crossed into the stimulated chamber, an Arduino microcontroller (www.arduino.cc) drove continuous yellow laser stimulation at 15mW or blue light stimulation at 20Hz (20% duty cycle), measured at 15mW when continuous. Pulsed blue light stimulation was achieved using a master 8 programmable pulse generator (A.M.P.I). Stimulation ceased when the animal returned to the non-stimulated chamber. Experiments were run without user interference and automatically terminated after 20 min.

#### Fiber photometry

##### Surgical procedures

For fiber placement for MHb-targeted fiber photometry experiments, AAV1-EF1a-DIO-GCaMP6f was injected in the MHb as described above and a fiber optic cannula (Doric Lenses, MFC_400/430–0.48_3.5mm_MF2.5(8 mm)_A45) was placed above the habenula (AP: −1.4, ML: ^+^/−0.5, DV: 2.85–2.95). Tips of the angled fiber optics were positioned on the lateral side of the target location. Cannulas were cemented in place with Metabond (Parkell) and animals were allowed to recover for at least 2 weeks. Of note, we found some serotype tropism for medial or lateral subregions of the habenula, and use AAV1 for all MHb-targeted injections. (Figure 3H). For LHb-targeted injections, AAV8-EF1a-DIO-GCaMP6f was injected in the LHb at AP: −1.4, ML: ^+^/− 0.45, DV: −3.05 and a fiber optic cannula (Doric Lenses, MFC_400/430–0.48_3.5mm_MF2.5(8 mm)_A45) was placed above the habenula (AP: −1.4, ML: ^+^/− 0.55, DV: 2.85–2.95).

##### Recording

Fiber photometry signals were recorded with a fiber optic patch cord (Doric Lenses, MFP_400/430/1100–0.48_5m_FC-MF2.5) coupled to the animals with a ceramic or brass sleeve. GCaMP was illuminated with LEDs emitting 405 and 490nm light, modulated with a lock-in amplifier, using the optical setup described (Lerner et al., 2015). Fluorescence was captured by a photodetector and processed with an RZ50 digital acquisition system (TDT Instruments) and Synapse Software (TDT instruments). Photometry data was synced to behavioral data using TTL outputs from Med Associates operant boxes, including trial starts, nose pokes, and entry into the reward port. Photometry data was analyzed offline using MATLAB. We would like to note that due to the low frequency of withheld:-withheld trial types, data is pooled from stages 4–6 during training for Figure S4B–E.

#### Reward history-guided decision-making

##### Surgical procedure

Mice were anesthetized with isoflurane and stereotaxic injections were used to deliver 2 viruses bilaterally. In the medial habenula, (AP: −1.4mm, ML ^+^/− 0.3mm, DV, −3.05mm) 200–300 nL of AAV1-nEF-Cre^ON^Flp^OFF^-eNpHR3.0-YFP at 5 × 10^12^ was delivered at a rate of 10 nL/min and 200nL of AAV8-Ef1a-FlpO at 7 × 10^11^ was delivered to the lateral habenula (AP: −1.4mm, ML ^+^/− 0.55mm, DV, −3.05mm). A dual core fiber optic cannula (ThorLabs CFM32L20) was cut to 3.5mm in length and inserted at AP −1.4 to a depth of DV −2.7. The fiber optic cannula and a custom headbar were cemented in place and the animal was allowed to recover.

##### Behavioral training

Two weeks after surgery, animals were water restricted and acclimated to head fixation. Behavioral shaping included several stages. Animals first learned to lick to both ports to receive water and rewarded licks resulted in an auditory cue. Next, a 1s pre-cue period was imposed during which all licks resulted in a premature termination of the trial. Upon auditory cue onset, licks at either port resulted in a water reward. After premature licking subsided, animals were forced to alternate between both lick ports in a block structure to reduce lick side bias. To encourage switching between sides in animals will side biases, small (2ul) rewards were delivered at the “correct” port after an “incorrect” port lick. After behavioral shaping, animals were moved to a block structure task. In each block, one lick port had a reward probability of 0.9 and the other a reward probability of 0.1. Blocks switched when animals had performed a minimum of 15 ^+^/− 3 “correct” licks at the high p_reward_ port. For the first 3 block switches in a session on a given day, a small reward was delivered on the new high p_reward_ port to prevent perseverative behavior toward one lick port. These first three block switches were not included in the analysis.

##### Optogenetic inhibition

Continuous 594 nm light was delivered bilaterally through a dual core patch cord (ThorLabs BFY32FL1) at 15mW combined power across both bilateral fibers. A subset of block switches were illuminated. On those blocks, any rewarded trials on trials 1–15 after the block switch were stimulated for 2s after the first rewarded lick.

#### Histology

##### Procedure

Following fiber photometry, optogenetic inhibition, and DIO-YFP experiments (Cre line validation and Serotype Testing), animals were deeply anesthetized, transcardially perfused with 4% PFA and postfixed overnight at 4C. Brains were sectioned at 50 μm thickness near the site of cannula placement, permeabilized in 0.1% TX100, and incubated overnight at 4C in anti-GFP antibody (ThermoFisher Cat # A-21311 or Cat# A-31852; 1:3000) to stain GCaMP expressing neurons. Sections were washed in PBS with 0.1% TX100 and imaged on a Leica SP5 Confocal Microscope.

#### Endoscopic two-photon calcium imaging

##### Surgery

Mice were anesthetized with 5% isoflurane and then maintained at surgical plane with 1–2% isoflurane. After betadine and ethanol cleaning, skin covering the dorsal aspect of the skull was made, and a 2 mm craniotomy was made above the injection side. After bleeding was stopped, a 36 gauge beveled needle attached to a nanoinjector Hamilton syringe was inserted into the medial habenula [+0.3 M/L, −1.4 A/P, −2.95 D/V]. 300 nL of AAV1-Ef1a-DIO-H2B-GCaMP6f was injected slowly (25 nL/min) (#3560 from Stanford Viral Vector core, 1.2 × 10^12^, undiluted). Ten minutes after injection ended, the syringe was removed. A 600 μm GRIN (Inscopix) was slowly (100 mm/min) inserted above the injection site [^+^0.3 M/L, −1.4 A/P, −2.60 D/V], and then cemented to the skull. A custom made head bar was cemented to the skull before covering the lens with wax paper and then Kwik-Cast (World Precision Instruments) for protection. Animals recovered in a warmed cage and then were individually housed. Two weeks later, animals that showed potential GCaMP expression were trained on this behavior.

##### Head-fixed behavior

A head-fixed behavioral setup was constructed using Bpod (Sanworks) and custom-fabricated parts. Each trial began with a 0.5 s pre cue period, in which a lick aborted the trial to the next ITI. If no premature licks occurred, a cue light was illuminated for 1 s, during which the animal could respond by licking an optical lickport (Sanworks). Timely response produced a 10–15 ul reward, which was followed by a 7–9 s ITI.

##### Imaging

Two photon imaging was conducted on a standard Neurolabware two photon microscope (8KHz resonance scanning, 512 lines per frame at 30 Hz/volume) tuned to 920 nm with a 16× Nikon dipping objective (NA = 0.8, WD – 3.0). Three planes were captured per imaging session. Imaging frames were aligned to trial start via TTL pulse.

#### Electrophysiology

##### Surgery and head-fixed behavior

The surgical procedure and the head-fixed behavior were mostly similar with those for two-photon calcium imaging experiments. For ChRmine expression, 300 nL of AAV1-nEF-DIO-ChRmine-oScarlet at 2 × 10^12^ vg/ml was injected to MHb [AP −1.4 mm, ML +/−0.3 mm, DV −2.95 mm]. For MHb-specific ChRmine expression, 300 nL of AAV1-nEF-Cre^On^Flp^Off^-ChRmine-oScarlet at 3 × 10^12^ vg/ml was injected to MHb, and 300 nL of AAV8-Ef1a-FlpO at 1 × 10^12^ vg/ml was injected to LHb [AP −1.4 mm, ML +/− 0.5 mm, DV −2.95 mm]. Injection speed was 25 nL/min and we waited at least 10 min post-injection before retracting syringes. A custom made headplate was cemented to the skull, and a reference electrode (ED1058-ND, Digi-key) was implanted away from the injection sites to just touch the brain surface. Then the rest of the skull was covered by “clear-skull cap”, i.e., layers consisting of cyanoacrylate adhesive (Krazy Glue) and clear nail polish (72,810, Electron Microscopy Sciences) (Zengcai Guo et al., Neuron, 2014; Allen, Kauvar et al. Neuron, 2017). Upon recovery for at least 3 days, the animals were water-restricted for at least 5 days before training onset. Then we trained the animals with 6 ul of plain water per rewarded trial. The durations of pre-cue, cue, and ITI epochs were 3, up to 2, and 4–6 s, respectively. Licking was detected by either optical (Sanworks) or electrical (comparator circuit, Janelia Research Campus) lickport.

##### In vivo *electrophysiology*

We used Neuropixels 2.0 probes (Steinmetz et al., 2021) lti-probe manipulator system (New Scale Technologies) and controlled by the SpikeGLX software (Janelia Research Campus), for the acute *in vivo* extracellular electrophysiological recordings acquired at 30 kHz. Prior to recording sessions, we performed craniotomy and durotomy, and the craniotomies were kept moist by frequent application of PBS and were covered by silicone elstomers (Qwik-Cast, World Precision Instruments) between recordings. For MHb-targeted recordings, a relatively large craniotomy (~2.5 mm diameter) was made on top of the midline at AP −1.4 mm. A 4-shank probe was inserted along an oblique (~10 deg) direction at slow speed (down to 200 μm/min near the target depth). While we typically targeted the probe tip to ~3500 μm from the brain surface, we decided the final depth on-the-fly by inferring the probe position relative to brain structures using the following heuristics. First, dentate gyrus shows high LFP signals and scarce spikes, and the spiking structure right below is habenula. Second, because MHb is right next to the third ventricle with no spikes, by examining overall spike rates of the individual shanks (separated ~250 μm from each other) approximate mediolateral position could be inferred. The probe was lowered ~100 μm past the final depth and then retracted to the final depth. Then we waited at least 15 min prior to recording for stabilization. The sites at the bottom ~700 μm of all shanks were used for recording. For septum-targeted recording, a small craniotomy (~1 mm) was made right next to the midline at AP +0.5 mm. Either a single- or 4-shank probe was inserted in a similar manner, typically targeting ~5000 μm from the brain surface. Bottom ~3000 μm of one shank was used for recording. The probes were cleaned with trypsin (Sigma-Aldrich) between recording sessions.

##### Transcranial optogenetics

For MHb recordings combined with transcranial optogenetics using ChRmine, we used a fiber-pigtailed 637 nm diode laser (OBIS, Coherent) driven by a voltage input (Bpod analog output module, Sanworks). The laser light was guided by a relayed 400 μm core, 0.39 NA optical fiber (M82L01, Thorlabs) positioned several millimeters above the skull by another manipulator arm. The space between the fiber tip and the skull was filled with PBS to improve coupling of stimulation light. Square pulses with ~800 mW/mm^2^ peak irradiance (at the fiber tip) and varying temporal parameters were used during or after behavioral recordings for perturbation and optotagging experiments, respectively. For transient perturbation, we used 10 ms width pulses at 30 Hz for 0.5 s per trial. For optotagging, we used 30 trials of 10 ms width pulses preceded by at least 1 s no-light periods.

## QUANTIFICATION AND STATISTICAL ANALYSIS

### In situ hybridization

To determine overlap of *Tac1, Th, Calb1*, and *ChAT* gene expression, confocal images were acquired on a Leica SP8 using Alexa 488, 514, 555, and 647 fluorophores attached to HCR hairpins, as well as DAPI staining to stain all cells. Using a FIJI macro, all DAPI labeled cells were hand-annotated, and then the corresponding fluorescence channels were scored for the presence or absence of each mRNA transcript.

### Elevated plus maze

Positional data was analyzed with BioObserve software. Fiber photometry signals were downstampled to the frame rate of the camera (10 fps). To generate heat maps, xy positions were binned every 1/3 cm and the mean of *Z*-scored photometry data was calculated for each bin.

### Behavioral video analysis

Animals were recorded while performing a 3-Choice Serial Reaction Time Task using commercially available USB web cameras. Frames were captured at 30 Hz with 1280 × 720 pixel resolution. Video analysis was performed with the open source software DeepLabCut (Mathis et al., 2018), post experimentation. Approximately 5 percent of frames per video were extracted to represent the diversity of animal poses during behavior, and manually labeled for nose, the center of implant, the back of implant, center of body mass, and tail base. Extracted and labeled frames were used to train a neural network for >100,000 iterations. The trained network was then used to analyze and estimate poses for the remaining frames. The x and y coordinates generated for the center and back of implant were used to create a vector indicating the head angle and movement of the animal. Head angle and movement were then aligned to fiber photometry data to fluorescence peaks correlated to behaviorally relevant periods.

### Fiber photometry

Fiber photometry data was collected using Synapse software (TDT Instruments). Timestamps were acquired from Med Associates operant training boxes (sampled at 10ms) and synced with the fiber photometry data in the Synapse Software. Raw photodetector measurements were processed offline in MATLAB (Lerner et al., 2015). Briefly, data from the control 405nm channel was downsampled to 60Hz and low pass filtered, and upsampled to the original sampling frequency (1017Hz). The smoothed control signal was fit to the GCaMP signal, and then subtracted from the GCaMP signal. The resulting value is reported as ΔF. For ΔF/F measurements, baseline rig fluorescence was measured without the animal subject and subtracted from the mean fluorescence during a session to determine the denominator F. For *Z*-score measurements, *Z*-scores were calculated using the entire behavioral session, from the start of the first trial to 10 s after the end of the last trial. For comparisons of timeseries across animals and genotypes or for visualization, we use *Z*-score to reduce animal to animal variability in viral expression. However, when reward statistics change across sessions (e.g., Figure 7K), we use ΔF/F to avoid *Z*-scoring artifacts. For within-animal, trial-wise comparisons, we use ΔF/F measurements.

### Reward history-guided decision-making

To reduce behavioral performance variability across days, only sessions where animals choice for the high reward port increased to >0.5 for trials on 10–15 after the block switch (across both stimulated and unstimulated transitions). To analyze block transitions (Figure S4F), the fraction of trials in which animals chose the new high p_reward_ port was calculated for light block and no light blocks. In light blocks, only rewarded licks in the new high p_reward_ port receive light illumination (0–2s after the first lick). The first lick to the high p_reward_ port can vary across animals and blocks, thus, the time of the first illumination also varies. To determine the effect of inhibition during reward at trial_t_ on the choice at trial_t+1_, the fraction of “stay” choices was calculated by taking all rewarded trials during trials_t=1–15_ of block switches and looking at the fraction of high p_reward_ port choices on trail_t+1_ (choices to stay). This was analyzed separately for trial_t_ = no light and trial_t_ = light. The response rate was calculated for this same set of trials_t=1–15_ by calculating the number of trials where the animals licked to either port, divided by the total number of trials. There were very few omissions during this task.

### Histology

Confocal images were analyzed in FIJI and quantified in MATLAB. Boundaries of the MHb and LHb were manually annotated for each section using a DAPI counterstain. In sections containing, or immediately adjacent to the fiberoptic cannula, cells expressing GCaMP were manually annotated. The small size, dense packing, and neuropil staining made automated cell counting unreliable in this format. For fiber photometry analyses, cells were determined to be in the collection cone of the fiber optic if they were <200 μm away from the face of the fiber, taking into account the NA of the fiber.

### Endoscopic two-photon calcium imaging

Images were extracted to tiff stacks using custom software. Image stacks were motion corrected using the FIJI plug in moco, and then downsampled to 256×256 pixels. CNMF (Pnevmatikakis et al., 2016) was run on these processed stacks to extract spatial footprints and time series for individual cells.

### Electrophysiology

#### Spike sorting

The electrophysiological traces were processed by a custom Python-based pipeline, with preprocessing (CatGT), spike sorting (Kilosort 2.5; ), waveform calculation (C_Waves), quality control metrics calculation (quality_metrics), and activity-behavior synchronization (TPrime) modules, initially written by the Allen Institute for Brain Science and Jennifer Colonell (Janelia Research Campus), and the sorted clusters were manually refined using Phy (Cyrille Rossant, International Brain Laboratory). We considered the units with <0.5 inter-spike interval violation metric (Hill et al., 2011; Siegle et al., 2021) single units, or “neurons”, and analyzed the neurons with >0.2 Hz firing rates.

#### Transcranial optogenetics

Prior to the spike sorting pipeline, light artifacts in the raw traces were computationally removed by subtracting trial-averaged traces for individual channels. To determine the optotagged single units, we used stimulus-associated spike latency test based on firstspike latency distributions (Kvitsiani et al., 2013; Kim et al., 2016).

#### Atlas registration

The imaging data were destripped and stitched by an accompanying software package (Swaney et al., 2019), obtaining full 3D volumes. Each autofluorescence volume was registered to a standard 3D mouse brain atlas (Allen Institute Common Coordinate Framework; CCFv3), and the corresponding transform was applied to all channels. For registration, we first used a series of automated rigid and non-rigid registration algorithms implemented by Elastix (elastix: a toolbox for intensity based medical image registration IEEE Transactions on Medical Imaging, Klein et al., 2010; Ye et al., 2016) and then manually adjusted the control points for further refinement (Swaney et al., 2019). For tracing the Neuropixels insertion tracks, we imported the registered volumes to Lasagna (OpenSerialSection) and then manually clicked on the visualized CM-DiI or DiD (spectrally and spatially distinguishable) tracks to obtain several coordinates along each track. These coordinates and the corresponding electrophysiological features (e.g., firing rates and LFPs over channels and time) were imported to a Python-based GUI for final registration. Specifically, several heuristics (e.g., no spikes in the ventricles, high LFPs in the dentate gyrus, or similar depths of individual shanks for the 4-shank probes) were utilized to find brain-region boundaries along the initial tracks, which determined to coordinates of the individual channels and thus of the single units recorded there. These coordinates determined the brain region label for each single unit based on the annotations in the CCFv3. Recorded brain regions were grouped based on CCFv3 hierarchy into the following 15 structures for downstream analyses: MHb (medial habenula), lateral habenula (LHb), LAT (lateral group of the dorsal thalamus), MED (medial group of the dorsal thalamus), MTN (midline group of the dorsal thalamus; includes PVT, paraventricular nucleus of the thalamus), ILM (intralaminar nuclei of the dorsal thalamus), DORsm (thalamus, sensorymotor cortex related), MB (midbrain), HIP (hippocampus), MSC (medial septal complex), TRS (triangular nucleus of septum), LSX (lateral septal complex), STR (striatum), BST (bed nuclei of the stria terminalis), and HY (Hypothalamus).

### Data analysis

Task-modulated neurons were identified by paired, two-sided Wilcoxon signed rank test of average firing rates for 1 s pre-cue baseline and four sequential 1 s windows from cue onset, separately for each trial type. A neuron was deemed task-modulated if any of these windows showed significant changes from the baseline (p < 0.01 after FDR correction across all neurons and windows using the Benjamini-Hochberg correction).

Time to baseline recovery for individual neurons upon transient optogenetic perturbation was estimated by paired, two-sided Wilcoxon signed rank test of average firing rates for 2 s pre-perturbation baseline and sequential 0.5 s windows from perturbation onset. We defined the recovery time as the time point such that the three consecutive windows starting from this time point show no significant changes from the baseline (p < 0.01 after FDR correction).

Individual neurons or populations were classified by ramping characteristics based on linear regression from trial index to average firing rate for 2 s pre-cue baseline. Two-sided p value for the null hypothesis that the slope is zero was calculated by estimating confidence interval of the slope. The significance threshold was p < 0.05, and the ramping direction was determined by the sign of the slope.

For quantifying trial-type-dependent within-trial population dynamics and integration, we first subtracted average firing rate for 2 s baseline for each trial to remove the baseline changes across trials. Then hierarchical bootstrap was used to combine data from multiple levels (Saravanan, Berman, Sober. Application of the hierarchical bootstrap to multi-level data in neuroscience. Neuron Behav Data Anal Theory. 2020). Each resampled dataset was constructed by randomly selecting a session, randomly selecting a neuron in the selected session, and randomly selecting a trial for the selected neuron, all with replacement, matching the size of the original dataset. Mean firing rate dynamics was calculated for each resampled dataset. After 100 iterations of this procedure, mean and SEM were calculated as mean and SD of the resampled means, respectively. For integration, we considered the baseline-subtracted average firing rate for the final 2 s window (5–7 s from cue or perturbation onset; right before the next event onset for the shortest ITI). The one-sided p values for the null hypothesis that this firing rate change, or integration along the total activity mode, is zero were calculated as the fraction of resampled means that are larger or smaller than zero. The significance threshold was p < 0.05.

### Dynamical systems modeling

We modified the original version of LFADS (Pandarinath et al., 2018a) to handle two-photon Ca^2+^ imaging data. Specifically, the Poisson distribution modeling discrete spikes was replaced by the Gaussian distribution modeling continuous fluorescence traces, and the corresponding likelihood function was changed accordingly. In addition, the processing of input data by the encoder RNN was set to be forward-directional in time only, and causal; that is, when decoding a time point *t* in a trial, the trLFADS generator only received information up to and including time point *t*. We made the architecture causal to aid interpretation of the trLFADS inferred inputs; this does not imply qualitative differences between the bidirectional versus unidirectional processing in time. For each session, the odd and even trials were split into training and validation sets respectively, to optimize the hyperparameters used in downstream analyses. We trained 4 total trLFADS models: one for each of two mice, and one for each Tac1^+^ and TH^+^ populations. Hyperparameters were as follows: number of units in the generator: 256; number of latent factors: 32; dimensionality of the inferred input: 1; number of units in the encoder: 256; number of units in the controller: 256; size of time bins: 200 ms; L2 penalty: 10^−9^ (Tac1^+^) or 10^−3^ (TH^+^); dropout keep rate: 0.98 (Tac1^+^) or 0.9 (TH^+^).

The fixed points were identified by minimizing of the trained generator RNN using gradient-based optimization (Barak et al., 2013). The input to the generator during optimization was the trial-averaged pre-cue input, which was close to zero. The squared speed thresholds were 0.0003 and 0.001 for Tac1^+^ and TH^+^, respectively.

The targeted dimensionality reduction (Mante et al., 2013) was based on three directions defined as follows. The total activity mode was defined as a vector of all 1s; the condition independent mode was defined as average activity difference between 1 s reward period and 1 s pre-cue period; the line attractor mode was defined as the first principal component of the relevant fixed points. The three vectors were orthonormalized using the above order using the Gram-Schmidt process before projection.

For the in silico dynamics experiments, we ran the trained generator RNN forward in time with trial-averaged inferred inputs (between −1 s before and 3 s after the cue onset) of either rewarded or unrewarded trials, sampled from a Bernoulli distribution of given reward probability. Between two consecutive trials 1 s relaxation time with zero effective input was given to address the inter-trial interval. We ran up to 10 rewarded trials per session and projected the trajectory to the total activity mode in order to predict fiber photometry. We simulated and averaged across 1,000 sessions, with random initial conditions on the line attractor, for each reward probability. All code was written in Python 3 with Jax for auto-differentiation (Frostig et al., 2018) and is available at https://github.com/google-research/computation-thru-dynamics.

## Supplementary Material

Table S1

Video S1

Video S2

4

## Figures and Tables

**KEY RESOURCES TABLE:** 

REAGENT or RESOURCE	SOURCE	IDENTIFIER

Antibodies		

anti-GFP antibody	Thermo Fisher Scientific	Cat# A-21311; RRID: AB_221477
anti-GFP antibody	Thermo Fisher Scientific	Cat# A-31852; RRID: AB_162553

Bacterial and virus strains		

AAV1-EF1a-DIO-GCaMP6f	Stanford Viral Vector Core	custom prep
AAV1-EF1a-DIO-YFP	Stanford Viral Vector Core	custom prep
AAV1-hSyn-YFP	Stanford Viral Vector Core	custom prep
AAV5-hSyn-YFP	UNC Viral Vector Core	custom prep
AAV8-hSyn-YFP	Stanford Viral Vector Core	custom prep
AAVdj-hSyn-YFP	Stanford Viral Vector Core	custom prep
AAVdj-hSyn-GCaMP6m	Stanford Viral Vector Core	custom prep
AAV8-EF1a-DIO-GCaMP6m	Stanford Viral Vector Core	custom prep
AAV1-EF1a-DIO-H2B-GCaMP6f	Stanford Viral Vector Core	custom prep
AAV1-Ef1a-DIO-ChRmine-oScarlet	Stanford Viral Vector Core	custom prep
AAV1-nEF-Cre^On^Flp^Off^-ChRmine-p2A-oScarlet	Stanford Viral Vector Core	custom prep
AAV1-nEF-Cre^On^Flp^Off^-eNpHR3.0-YFP	Stanford Viral Vector Core	custom prep
AAV8-Ef1a-FlpO	Stanford Viral Vector Core	custom prep

Chemicals, peptides, and recombinant proteins		

methacryloxypropyltrimethoxysilane	GE Healthcare	17-1330-01
poly-L-lysine	Sigma-Aldrich	P6407
Glass Bottom Plates	MatTekq	P12G-1.5-14-F
Micro coverglass	EMS	72,226-01
OTC	Fisher	23-730-571
16% PFA	EMS	15710-S
PBS	Gibco	70,011–044
Triton X-100	Sigma-Aldrich	93,443
OminiPur Formamide	Calbiochem	75-12-7
20×SSC buffer	Sigma-Aldrich	S6639
dNTP mix	Invitrogen	100,004,893
Tween 20	Calbiochem	655,206
SUPERase	Invitrogen	AM2696
RVC	New England Biolabs	S1402S
Salmon Sperm	Invitrogen	AM9680
T4 DNA ligase	Thermo Scientific	EL0011
Phi29 DNA polymerase	Thermo Fisher Scientific	EP0094
BSA	New England Biolabs	EP0094
BSPEG9	Thermo Fisher Scientific	21,582
Phi29 DNA polymerase	Thermo Fisher Scientific	EP0094
Acrylic acid NHS ester	Sigma-Aldrich	A8060
Methacrylic acid NHS ester	Sigma-Aldrich	730,300
DMSO	Sigma-Aldrich	D12345
Acrylamide, 40%	BioRad	161-0140
Bis-acrylamide, 2%	BioRad	161-0142
Ammonium persulfate	Sigma-Aldrich	A3678
Tetramethylethylenediamine	Sigma-Aldrich	T9281
OminiPur SDS	Calbiochem	7991
Shrimp Alkaline Phosphatase	New England Biolabs	M0371L
DAPI	Molecular Probes	D1306
NeuroTrace Nissl Stain	Molecular Probes	N-21480
Gel Slick	Lonza	50,640
Proteinase K	Invitrogen	25,530,049
superfrost plus slides	Fisher Scientific	22-037-246
Hybrislips	EMS	7,032,962
Trimethroprim (TMP)	Sigma-Aldrich	92,131-1G
Heparin	Sigma-Aldrich	H4784-250MG
50× Denhardt’s solution	Sigma-Aldrich	D2532-5ML
Dextran Sulfate	Sigma-Aldrich	D6001
Fluorescent beads	Lumafluor	Red Retrobeads
Alexa 514-Labeled Hairpins	Molecular Technologies	B5-H1/H2-514
Alexa 555-Labeled Hairpins	Molecular Technologies	B2-H1/H2-555
Alexa 593-Labeled Hairpins	Molecular Technologies	B4-H1/H2-593
Alexa 647-Labeled Hairpins	Molecular Technologies	B1-H1/H2-647
Methanol	Sigma-Aldrich	322,415
Dichloromethane	Sigma-Aldrich	270,997
Ethyl cinnamate	Sigma-Aldrich	112,372
Trypsin	Sigma-Aldrich	59427C
CM-DiI	Thermo Fisher Scientific	V22888
DiD	Thermo Fisher Scientific	D7757
DID-DS	Thermo Fisher Scientific	D12730

Experimental models: Organisms/strains		

Th-Cre (Th^thm1(cre)Te^	Lindeberg	MGI:3056580
B_6_; 129S-*Tac1^tm1.1(cre)Hze^*	Jax	021,877
Tg(Chat-cre)GM24Gsat/Mmucd	MMRRC	#017269-UCD
B_6_.Cg-*Calb1^tm1.1(folA/cre)Hze^*/J	Jax	#023531
C57/Bl6	Jax	#000664

Oligonucleotides		

Calb1_01	IDT (for STARmap)	/5Phos/ACATTAGCCAACTCTACAATTCCTATAATTATTAATGAAACATACACTAAAGATA
Calb1_02	IDT (for STARmap)	/5Phos/ACATTATCAGCGTCGAAATGAAGCCAGAATTATTAATGAAACATACACTAAAGATA
Calb1_03	IDT (for STARmap)	/5Phos/ACATTAGGAAATTTTCCTGCACTGGTAATTATTAATGAAACATACACTAAAGATA
Calb1_04	IDT (for STARmap)	/5Phos/ACATTAATTTCATTTCCGGTGATAGCTAATTATTAATGAAACATACACTAAAGATA
Calb1_11	IDT (for STARmap)	TCTTCTGTGGGTAAGACGTGTAATGTTATCTT
Calb1_12	IDT (for STARmap)	CTTCCAGGTAACCACTTCCGTAATGTTATCTT
Calb1_13	IDT (for STARmap)	TGATTCCCTGGAATTTAAGATAATGTTATCTT
Calb1_14	IDT (for STARmap)	CTGTCCATATTGATCCACAAATAATGTTATCTT
Calb2_01	IDT (for STARmap)	/5Phos/ACATTAGAGCACAATCTCCAGGTCCTAATTATTACTGAAACATACACTAAAGATA
Calb2_02	IDT (for STARmap)	/5Phos/ACATTAGGTGGTGAGCTGTTGGATGTAATTATTACTGAAACATACACTAAAGATA
Calb2_03	IDT (for STARmap)	/5Phos/ACATTAATCCGTAGTATGGTCTGGGTGTAATTATTACTGAAACATACACTAAAGATA
Calb2_04	IDT (for STARmap)	/5Phos/ACATTAAGCCCACGTGCTGCCTGAAGCAAATTATTACTGAAACATACACTAAAGATA
Calb2_11	IDT (for STARmap)	TTACACGGGGGGCTCACTGCTAATGTTATCTT
Calb2_12	IDT (for STARmap)	AGGACATGACACTCTTCCTGTAATGTTATCTT
Calb2_13	IDT (for STARmap)	TGCCATCTCCATTTAAGTCAAATAATGTTATCTT
Calb2_14	IDT (for STARmap)	AAGCCTCCATAAACTCAGCGCTTAATGTTATCTT
Cartpt_01	IDT (for STARmap)	/5Phos/ACATTACCTTTCCTCACTGCGCACTGAATTATTAGTGAAACATACACTAAAGATA
Cartpt_02	IDT (for STARmap)	/5Phos/ACATTAAAGTTGCCGCCTTGGCAGCTAATTATTAGTGAAACATACACTAAAGATA
Cartpt_03	IDT (for STARmap)	/5Phos/ACATTAGCGTTTACTCTTGAGCTTCTAATTATTAGTGAAACATACACTAAAGATA
Cartpt_04	IDT (for STARmap)	/5Phos/ACATTAAGTAGCAGCAGGGCGGCGCCCAATTATTAGTGAAACATACACTAAAGATA
Cartpt_11	IDT (for STARmap)	ACAGCTTCCCGATCCTGGCTAATGTTATCTT
Cartpt_12	IDT (for STARmap)	AACATAGCGCCGGGAGCCCTAATGTTATCTT
Cartpt_13	IDT (for STARmap)	ACTTCTTCTCGTAGATCGGATAATGTTATCTT
Cartpt_14	IDT (for STARmap)	ACGGGCACCCAGCAAAGGTATAATGTTATCTT
Chat_01	IDT (for STARmap)	/5Phos/ACATTATTCGCTCCATTCAAGCTGCAAATTATTATTGAAACATACACTAAAGATA
Chat_02	IDT (for STARmap)	/5Phos/ACATTAGGACGCCATTTTGACTATCTAATTATTATTGAAACATACACTAAAGATA
Chat_03	IDT (for STARmap)	/5Phos/ACATTATCTCTCATGTCAACAAGGCTAATTATTATTGAAACATACACTAAAGATA
Chat_04	IDT (for STARmap)	/5Phos/ACATTAATTAATGACAACATCCAAGACAAATTATTATTGAAACATACACTAAAGATA
Chat_11	IDT (for STARmap)	GGGACTTGTCATACCAACGTAATGTTATCTT
Chat_12	IDT (for STARmap)	GGCAGGCGTTCATCCTCGTTAATGTTATCTT
Chat_13	IDT (for STARmap)	AGGCTGCCTCGAACTACAGATAATGTTATCTT
Chat_14	IDT (for STARmap)	TCACCCTCACTGAGACGGCGGTAATGTTATCTT
Elfn1_01	IDT (for STARmap)	/5Phos/ACATTAAGTCTGAGACGCTCCCAGATAATTATTAATGAACCATACACTAAAGATA
Elfn1_02	IDT (for STARmap)	/5Phos/ACATTACGTCGATGCAGTTGTTAATGAATTATTAATGAACCATACACTAAAGATA
Elfn1_03	IDT (for STARmap)	/5Phos/ACATTATACAGGTACTCGAGCTTGCTAATTATTAATGAACCATACACTAAAGATA
Elfn1_04	IDT (for STARmap)	/5Phos/ACATTACGTACTGGCGACTCTTGTCGGCAATTATTAATGAACCATACACTAAAGATA
Elfn1_11	IDT (for STARmap)	CCTTGTGTCTCCGTCGGCTTAATGTTATCTT
Elfn1_12	IDT (for STARmap)	GGTGGACTCGGACTTGAGGTAATGTTATCTT
Elfn1_13	IDT (for STARmap)	CGATGAGGTTGGCCTGTAGTAATGTTATCTT
Elfn1_14	IDT (for STARmap)	GGGGTATGAGTGCCGATGCTCTAATGTTATCTT
GAD2_01	IDT (for STARmap)	/5Phos/ACATTAGGACATCAGTAACCCTCCACAATTATTACTGAACCATACACTAAAGATA
GAD2_02	IDT (for STARmap)	/5Phos/ACATTACTCTAACCAGGAGAGCTGAAAATTATTACTGAACCATACACTAAAGATA
GAD2_03	IDT (for STARmap)	/5Phos/ACATTAAACGCGTAGTTGACATCCCCTTAATTATTACTGAACCATACACTAAAGATA
GAD2_04	IDT (for STARmap)	/5Phos/ACATTACTCCAGATTTTGCGGTTGGTCTAATTATTACTGAACCATACACTAAAGATA
GAD2_11	IDT (for STARmap)	AGCTTCCACTTGTGTTTCCTAATGTTATCTT
GAD2_12	IDT (for STARmap)	AGCTCTGCATCAGTCCCTCCTAATGTTATCTT
GAD2_13	IDT (for STARmap)	AGCAGGTCTGTTGCGTGCATAATGTTATCTT
GAD2_14	IDT (for STARmap)	TGTTTGGCAATGCGTCAAAATTTAATGTTATCTT
Gpr151_01	IDT (for STARmap)	/5Phos/ACATTAAACAAATGTCAAGCTCTTGGAATTATTAGTGAACCATACACTAAAGATA
Gpr151_02	IDT (for STARmap)	/5Phos/ACATTAATTGTCTTGTGCTGAAGGGAAATTATTAGTGAACCATACACTAAAGATA
Gpr151_03	IDT (for STARmap)	/5Phos/ACATTATAGGATGCATGTTTGGGTCTAATTATTAGTGAACCATACACTAAAGATA
Gpr151_04	IDT (for STARmap)	/5Phos/ACATTAGTTGCTGGAATTGGTGTCGGAATTATTAGTGAACCATACACTAAAGATA
Gpr151_11	IDT (for STARmap)	AAGCATGCTTTGGCTACCATAATGTTATCTT
Gpr151_12	IDT (for STARmap)	TGTTCCCAGGGTATAGGGTTAATGTTATCTT
Gpr151_13	IDT (for STARmap)	AGACCCACGTCCGTCTGTGTTAATGTTATCTT
Gpr151_14	IDT (for STARmap)	GCGAGCAAACGACTCGTTCATAATGTTATCTT
Htr5b_01	IDT (for STARmap)	/5Phos/ACATTACACGCCAATCAAGATCCCGAAATTATTATTGAACCATACACTAAAGATA
Htr5b_02	IDT (for STARmap)	/5Phos/ACATTAAAGGTTACTGTTGCTCGGCGAATTATTATTGAACCATACACTAAAGATA
Htr5b_03	IDT (for STARmap)	/5Phos/ACATTAGGACAACTCGCTCACCAGGCTAATTATTATTGAACCATACACTAAAGATA
Htr5b_04	IDT (for STARmap)	/5Phos/ACATTAATTCCATAAGAAAGTGGCAACGAATTATTATTGAACCATACACTAAAGATA
Htr5b_11	IDT (for STARmap)	GGGGATCCAACAAAGCACAATAATGTTATCTT
Htr5b_12	IDT (for STARmap)	CCACGAGTCTCCGCTTGTCTTAATGTTATCTT
Htr5b_13	IDT (for STARmap)	CCTAGCTGCCAACGTCGCCCATAATGTTATCTT
Htr5b_14	IDT (for STARmap)	AGGATAGTCACCAGAACTAGCTAATGTTATCTT
Oprm1_01	IDT (for STARmap)	/5Phos/ACATTAGCATGATGAAGGCGAAGATGAATTATTAATGAAGCATACACTAAAGATA
Oprm1_02	IDT (for STARmap)	/5Phos/ACATTACTTGAGTCGTAAGATCATCAGTAATTATTAATGAAGCATACACTAAAGATA
Oprm1_03	IDT (for STARmap)	/5Phos/ACATTAACCCCTGCCTGTATTTTGTGGTAATTATTAATGAAGCATACACTAAAGATA
Oprm1_04	IDT (for STARmap)	/5Phos/ACATTACCGTGGAGGGGTGTTCCCTAGTAATTATTAATGAAGCATACACTAAAGATA
Oprm1_11	IDT (for STARmap)	ACACAGTGATGATGAGGACCTAATGTTATCTT
Oprm1_12	IDT (for STARmap)	GGAGCCCGACAGCATGCGGACATAATGTTATCTT
Oprm1_13	IDT (for STARmap)	AGAACGTGAGGGTGCAATCTATTAATGTTATCTT
Oprm1_14	IDT (for STARmap)	GGTTAGTTCGATCCACTGTATTTAATGTTATCTT
Slc17a7_01	IDT (for STARmap)	/5Phos/ACATTAGACGTAAAGAAGCGCCTCCAAATTATTACTGAAGCATACACTAAAGATA
Slc17a7_02	IDT (for STARmap)	/5Phos/ACATTAACATTATGTGACGACTGCGCAATTATTACTGAAGCATACACTAAAGATA
Slc17a7_03	IDT (for STARmap)	/5Phos/ACATTATTCAAAGTAGGCGGGCTGAGAGAATTATTACTGAAGCATACACTAAAGATA
Slc17a7_04	IDT (for STARmap)	/5Phos/ACATTAAGGGTGGAGGTAGCCACAATGGAATTATTACTGAAGCATACACTAAAGATA
Slc17a7_11	IDT (for STARmap)	ATGATGGCATAGACGGGCATTAATGTTATCTT
Slc17a7_12	IDT (for STARmap)	AGCTTTCGCACGTTGGTAGTTAATGTTATCTT
Slc17a7_13	IDT (for STARmap)	GCTGATCTCAAAGCCGAACACTTAATGTTATCTT
Slc17a7_14	IDT (for STARmap)	GCTGCTGAAGGGATCAACATGTTAATGTTATCTT
Sst_01	IDT (for STARmap)	/5Phos/ACATTACCATTGCTGGGTTCGAGTTGAATTATTAGTGAAGCATACACTAAAGATA
Sst_02	IDT (for STARmap)	/5Phos/ACATTACCAGAAGAAGTTCTTGCAGCAATTATTAGTGAAGCATACACTAAAGATA
Sst_03	IDT (for STARmap)	/5Phos/ACATTACCAGTTCCTGTTTCCCGGTGAATTATTAGTGAAGCATACACTAAAGATA
Sst_04	IDT (for STARmap)	/5Phos/ACATTAGACGGAGTCTGGGGTCCGAGGGAATTATTAGTGAAGCATACACTAAAGATA
Sst_11	IDT (for STARmap)	AGCTTTGCGTTCCCGGGGTTAATGTTATCTT
Sst_12	IDT (for STARmap)	CTAACAGGATGTGAATGTCTTAATGTTATCTT
Sst_13	IDT (for STARmap)	AGCTCTGCCAAGAAGTACTTTAATGTTATCTT
Sst_14	IDT (for STARmap)	CGCCAGAGACTTCTGCAGAAATAATGTTATCTT
Sstr4_01	IDT (for STARmap)	/5Phos/ACATTAAAGCACTGCGCGACACAGCAAATTATTATTGAAGCATACACTAAAGATA
Sstr4_02	IDT (for STARmap)	/5Phos/ACATTAGCACGAGCCTAGTGATCTTCAATTATTATTGAAGCATACACTAAAGATA
Sstr4_03	IDT (for STARmap)	/5Phos/ACATTAGATGACCAGGGCGTTTCCCAAATTATTATTGAAGCATACACTAAAGATA
Sstr4_04	IDT (for STARmap)	/5Phos/ACATTAAGAACCGGGAGTAGGAAGCCCAAATTATTATTGAAGCATACACTAAAGATA
Sstr4_11	IDT (for STARmap)	ATGTTCAGGCCGTCCACGCTAATGTTATCTT
Sstr4_12	IDT (for STARmap)	AAAGACGGTCACCACCATTTAATGTTATCTT
Sstr4_13	IDT (for STARmap)	TGGCATAGCGTAGGATCACGTAATGTTATCTT
Sstr4_14	IDT (for STARmap)	AGCAGGTAGCATAATCCGATGGTAATGTTATCTT
Syt10_01	IDT (for STARmap)	/5Phos/ACATTAGTCGTCTTTTCTGTTGCCCTAATTATTAATGAATCATACACTAAAGATA
Syt10_02	IDT (for STARmap)	/5Phos/ACATTATTCGACACACGCCTATGACCTAATTATTAATGAATCATACACTAAAGATA
Syt10_03	IDT (for STARmap)	/5Phos/ACATTATTTCCATACTGTGGCTTCCCTAATTATTAATGAATCATACACTAAAGATA
Syt10_04	IDT (for STARmap)	/5Phos/ACATTACTCGTTCCAATGGTCTCTCCCAAATTATTAATGAATCATACACTAAAGATA
Syt10_11	IDT (for STARmap)	AGTTTCCCACAGGTCTTGATAATGTTATCTT
Syt10_12	IDT (for STARmap)	AGACCCTCAGCGTCTAGTCCTAATGTTATCTT
Syt10_13	IDT (for STARmap)	TCTGTGGTAGCACAGTGGATATAATGTTATCTT
Syt10_14	IDT (for STARmap)	TGGCTTTCGGTGATAGGCCAGCTAATGTTATCTT
Tac1_01	IDT (for STARmap)	/5Phos/ACATTAGCGATTCTCTGCAGAAGATGAATTATTACTGAATCATACACTAAAGATA
Tac1_02	IDT (for STARmap)	/5Phos/ACATTACAAAGAACTGCTGAG GCTTGAATTATTACTGAATCATACACTAAAGATA
Tac1_03	IDT (for STARmap)	/5Phos/ACATTAATCGCGCTTCTTTCATAAGCAATTATTACTGAATCATACACTAAAGATA
Tac1_04	IDT (for STARmap)	/5Phos/ACATTACCCATTAGTCCAACAAAGGAATAATTATTACTGAATCATACACTAAAGATA
Tac1_11	IDT (for STARmap)	GCTGAGGCTTGGGTCTTCGTAATGTTATCTT
Tac1_12	IDT (for STARmap)	ATCCCGCTTGCCCATTAATTAATGTTATCTT
Tac1_13	IDT (for STARmap)	ACGTCTTCTTTCGTAGTTCTTAATGTTATCTT
Tac1_14	IDT (for STARmap)	GCCACAGAATTTAAAGCTCTTTTAATGTTATCTT
Tac2_01	IDT (for STARmap)	/5Phos/ACATTAAGCCGCAAACAGCATGGCGCAATTATTAGTGAATCATACACTAAAGATA
Tac2_02	IDT (for STARmap)	/5Phos/ACATTACGGTGGGAGTGTCTGGTTGGCAATTATTAGTGAATCATACACTAAAGATA
Tac2_03	IDT (for STARmap)	/5Phos/ACATTATGTCACGTTTCTGTGGAAGTGAATTATTAGTGAATCATACACTAAAGATA
Tac2_04	IDT (for STARmap)	/5Phos/ACATTATCTCCGAAGCAGGGACGGAGGCAATTATTAGTGAATCATACACTAAAGATA
Tac2_11	IDT (for STARmap)	AGCCAAGCTGAGGGCGAGGATAATGTTATCTT
Tac2_12	IDT (for STARmap)	GGGGTGTTCTCTTCAACCACTAATGTTATCTT
Tac2_13	IDT (for STARmap)	AGTCCCACAAAGAAGTCGTGTAATGTTATCTT
Tac2_14	IDT (for STARmap)	AGAGACAGGGCGGCTGTCGTAGTAATGTTATCTT
Th_01	IDT (for STARmap)	/5Phos/ACATTAGAAGTGAGACACATCCTCCAAATTATTATTGAATCATACACTAAAGATA
Th_02	IDT (for STARmap)	/5Phos/ACATTACTCGAATACCACAGCCTCCAAATTATTATTGAATCATACACTAAAGATA
Th_03	IDT (for STARmap)	/5Phos/ACATTAGGCTTCAAATGTCTCAAACACTAATTATTATTGAATCATACACTAAAGATA
Th_04	IDT (for STARmap)	/5Phos/ACATTAGAGGCATAGTTCCTGAGCTTGTAATTATTATTGAATCATACACTAAAGATA
Th_11	IDT (for STARmap)	AAGCCAGTCCGTTCCTTCATAATGTTATCTT
Th_12	IDT (for STARmap)	ACAGCATTTCCATCCCTCTTAATGTTATCTT
Th_13	IDT (for STARmap)	CCGGGTCTCTAAGTGGTGGATTTAATGTTATCTT
Th_14	IDT (for STARmap)	ACAGAGAATGGGCGCTGGATACTAATGTTATCTT
Mus-Calb1-B4P1	IDT (for ISH)	cactctcaaactagccgctgcaccacgatggcagaatcccacctgcagtcat
Mus-Calb1-B4P2	IDT (for ISH)	agaaaagtagatatcgtgaatcattatagccgaaggactctaataaaaattt
Mus-Calb1-B4P3	IDT (for ISH)	aatagtaccaagtttggcattcttactaatagtagtagaacaaatcctataa
Mus-Calb1-B4P4	IDT (for ISH)	cgccgcgcccagctcagcctgcgcagccctctcgcccgaggttcgcgctccg
Mus-Calb1-B4P5	IDT (for ISH)	aagactgtggatgatacaaaactagcagagtacacagacctcatgctgaaac
Mus-Calb1-B4P6	IDT (for ISH)	ccagtgcaggaaaatttccttcttaaattccagggaatcaaaatgtgtggga
Mus-Calb1-B4P7	IDT (for ISH)	ggagggaagctgtaccgaacagaccttgctcttattctttctgctggagaca
Mus-Calb1-B4P8	IDT (for ISH)	tagagttggtgaccacaaccacttgctagtgatacattgtatctaaaaccat
Mus-Calb1-B4P9	IDT (for ISH)	aaatatcaacagttaattatggctttattctgaaacgatctccctagagatt
Mus-Calb1-B4P10	IDT (for ISH)	atgcctatatttccaagaagtctactgccagagagtatgaccatagcccatt
Mus-Calb1-B4P11	IDT (for ISH)	ctaaattattttcatgtgttccagatgacaattattctagtaaactgctgtt
Mus-Calb1-B4P12	IDT (for ISH)	tatttcatcaaaacttgtgtattctgtggattctatggttcatattgagatc
Mus-Calb1-B4P13	IDT (for ISH)	aggtttgggtcaggttggatttaagcactttttttcaattgttgttcataaa
Mus-Calb1-B4P14	IDT (for ISH)	atttgtttctaccccaaagtgttaattgtcatgtaatctgttatcaattagg
Mus-Calb1-B4P15	IDT (for ISH)	gcgcgaaagaaggctggattggagctatcaccggaaatgaaatcctttgtgg
Mus-Calb1-B4P16	IDT (for ISH)	caatatggacagagagatgatggaaaaataggaattgtagagttggctcacg
Mus-Calb1-B4P17	IDT (for ISH)	ttacccacagaagagaatttcttgctgctctttcgatgccagcaactgaagt
Mus-Calb1-B4P18	IDT (for ISH)	atcgaaaccgaggaacttaagaactttctaaaggacctactagagaaagcaa
Mus-Calb1-B4P19	IDT (for ISH)	tttgattcaaataatgacggaaagctggaactgacagagatggccaggttac
Mus-Calb1-B4P20	IDT (for ISH)	gagttcaataaggcttttgagttatatgatcaggatggcaacggatacatag
Mus-ChAT-B5P1	IDT (for ISH)	gcgtccaacgaggatgaacgcctgcctccaatcggcctgctgacgtcagacg
Mus-ChAT-B5P2	IDT (for ISH)	gctaggatgcctatcctggaaaaggtccccccaaagatgcctgtacaagctt
Mus-ChAT-B5P3	IDT (for ISH)	gaatactggctgaatgacatgtatctaaacaaccgcctggccctgccagtca
Mus-ChAT-B5P4	IDT (for ISH)	cgccgtctcagtgagggtgatctgttcactcagttgagaaagatagtcaaaa
Mus-ChAT-B5P5	IDT (for ISH)	atgagagacctctgtagttcgaggcagcctgctgaaggcaagccaccaacag
Mus-ChAT-B5P6	IDT (for ISH)	aaggaaagagctagaggcccaaccaagccaagcaatcttgactactcccact
Mus-ChAT-B5P7	IDT (for ISH)	ggtctttagaaacttaacctttctgcttctttcccagcaacacccagtggtg
Mus-ChAT-B5P8	IDT (for ISH)	gcgtaacagcccaggagagcaggtcggcagctctgctactctggattaagaa
Mus-ChAT-B5P9	IDT (for ISH)	agctgtgaggaggtgctggacttacctaagttgccagtgcccccactgcagc
Mus-ChAT-B5P10	IDT (for ISH)	aggaagagccaggccattgtgaagcggtttggggcccctggtggcctgggtg
Mus-ChAT-B5P11	IDT (for ISH)	tctagccctgctgtgatctttgctcggcagcacttccaagacaccaatgacc
Mus-ChAT-B5P12	IDT (for ISH)	ctaaggtttgcagccagcctcatctctggtgtgcttagctacaaggctctgc
Mus-ChAT-B5P13	IDT (for ISH)	ggcactggagacctcagtgacacacacagggccctccagctccttcatggtg
Mus-ChAT-B5P14	IDT (for ISH)	ggctgcagcttgaatggagcgaatcgttggtatgacaagtccctgcagtttg
Mus-ChAT-B5P15	IDT (for ISH)	aagaagctcgtccgagctgactcagtgagtgaactccctgctcccagaaggc
Mus-ChAT-B5P16	IDT (for ISH)	gccctccagctggcttactacaggctttaccagaggctggtgcccacctatg
Mus-ChAT-B5P17	IDT (for ISH)	tacacagtcatggccataaccggcatggccattgacaaccatcttctggcac
Mus-ChAT-B5P18	IDT (for ISH)	tataacccccagcctgaggccatcaccttctgcatctccagctttcacggct
Mus-ChAT-B5P19	IDT (for ISH)	gcttgttgctgctcccctatccttgggggctcacatgaagctggcatgttaa
Mus-ChAT-B5P20	IDT (for ISH)	atgagacccagcctggcttggaagcagcctgggtgggctgggagctccctct
Mus-Tac1-B2P1	IDT (for ISH)	gtgcgcacctgcggagcatccccgcggtctgaccgcaaaatcgaacatgaaa
Mus-Tac1-B2P2	IDT (for ISH)	aatcgatgccaacgatgatctaaattattggtccgactggtccgacagtgac
Mus-Tac1-B2P3	IDT (for ISH)	gatcaaggaggcaatgccggagccctttgagcatcttctgcagagaatcgcc
Mus-Tac1-B2P4	IDT (for ISH)	tttaaattctgtggcttatgaaagaagcgcgatgcagaactacgaaagaaga
Mus-Tac1-B2P5	IDT (for ISH)	ataatgtactgagacttcggtatttgactctatttgtatcctagcagcatgt
Mus-Tac1-B2P6	IDT (for ISH)	ctctcacaaaaggcataaaacagattcctttgttggactaatgggcaaaaga
Mus-Tac1-B2P7	IDT (for ISH)	taaataaacccctgaacgcactatctattcatcttcatctgtgtcagtgagc
Mus-Tac1-B2P8	IDT (for ISH)	gtaatttcagcaaagcacagtgatgaaggagctgtccaagcttggcagtgac
Mus-Tac1-B2P9	IDT (for ISH)	gcagagactcctgtgcgtctctctcacgctacccctggttctgctttcatgc
Mus-Tac1-B2P10	IDT (for ISH)	cctgtttcgtgactatatagagatgttttgaaaaagtttcaatgtaattctc
Mus-Tac1-B2P11	IDT (for ISH)	aagacccaagcctcagcagttctttggattaatgggcaagcgggatgctgat
Mus-Tac1-B2P12	IDT (for ISH)	cggacctgctccgctcctgcaccgcggccaaggagagcaaagagcgcccagc
Mus-Tac1-B2P13	IDT (for ISH)	cctcgtggccgtggcggtcttttttctcgtttccactcaactgtttgcagag
Mus-Tac1-B2P14	IDT (for ISH)	ctcagttgaaaaacaagtggccctgttaaaggctctttatggacatggccag
Mus-Tac1-B2P15	IDT (for ISH)	tgaacggtaaaataaaatgtgcgctatgaggaatgattatttatttaataac
Mus-Tac1-B2P16	IDT (for ISH)	ccaataagccttgtaattctaatgtggtgacctccccagaagtagaaattag
Mus-Tac1-B2P17	IDT (for ISH)	atgttgttgtgagtgaaaaactcaaaaaagaagtgtttattttttcatattg
Mus-Tac1-B2P18	IDT (for ISH)	agtctccaaagaaaggacccttctgtgagccagcgcaggcagctgctgctgg
Mus-Tac1-B2P19	IDT (for ISH)	gtcttcagtcattgtatgatgtgttgtgatagctaccattttaaataaaaga
Mus-Th-B1P1	IDT (for ISH)	ctcctcagttctgtgcgtcgggtgtctgacgatgtgcgcagtgccagagagg
Mus-Th-B1P2	IDT (for ISH)	gtactggacagtcctcacaccatccggcgctccttagagggggtccaggatg
Mus-Th-B1P3	IDT (for ISH)	tcagagcaggataccaagcaggccgaggctgtcacgtccccaaggttcattg
Mus-Th-B1P4	IDT (for ISH)	cggcggcagagtctcatcgaggatgcccgcaaggagcgggaggcagcagcag
Mus-Th-B1P5	IDT (for ISH)	gcagcagcggctgcggtagcctccgcggaacctgggaacccattggaggctg
Mus-Th-B1P6	IDT (for ISH)	gtattcgaggagagggatggaaatgctgttctcaacctgctcttctccttga
Mus-Th-B1P7	IDT (for ISH)	gccaaaatccaccacttagagacccggcctgcccagaggccactggcaggaa
Mus-Th-B1P8	IDT (for ISH)	ccccacctggagtactttgtgcgcttcgaggtgcccagtggcgacctggctg
Mus-Th-B1P9	IDT (for ISH)	aaggttccctggttcccaaggaaagtgtcagagttggataagtgtcaccacc
Mus-Th-B1P10	IDT (for ISH)	gaggtatacgccacgctgaagggcctctatgctacccatgcctgccgggaac
Mus-Th-B1P11	IDT (for ISH)	cgacccgtggccggtctactgtctgcccgtgattttctggccagtctggcct
Mus-Th-B1P12	IDT (for ISH)	cgtgtgtttcagtgcacacagtacatccgtcatgcctcctcacctatgcact
Mus-Th-B1P13	IDT (for ISH)	tatggagagctcctgcactccctgtcagaggagcccgaggtccgggcctttg
Mus-Th-B1P14	IDT (for ISH)	gtcaccaagtttgaccctgacctggacctggaccatccgggcttctctgacc
Mus-Th-B1P15	IDT (for ISH)	gcgtatcgccagcgccggaagctgattgcagagattgccttccaatacaagc
Mus-Th-B1P16	IDT (for ISH)	ctggaggctttccagcttctggaacggtactgtggctaccgagaggacagca
Mus-Th-B1P17	IDT (for ISH)	Cccgagccagactgctgccacgagctgctgggacacgtacccatgttggctg
Mus-Th-B1P18	IDT (for ISH)	ctgtgtaaacagaatggggagctgaaggcttacggtgcagggctgctgtctt
Mus-Th-B1P19	IDT (for ISH)	ccagacacagcagccgtgcagccctaccaagatcaaacctaccagccggtgt
Mus-Th-B1P20	IDT (for ISH)	cgtatccagcgcccattctctgtgaagtttgacccgtacaccctggccattg

Software and algorithms		

MedPC-IV	MedAssociates	https://www.med-associates.com/med-pc-v/
Viewer3	BioObserve	http://www.biobserve.com/behavioralresearch/
FIJI	OpenSource	https://ImageJ.net/ImageJ
MATLAB	Mathworks	https://www.mathworks.com/products/matlab.html
DeepLabCut	Mathis et al. (2018)	https://github.com/DeepLabCut
CNMF	Pnevmatikakis et al. (2016)	https://github.com/epnev/ca_source_extraction
SpikeGLX (and associated command-line tools: CatGT, TPrime, and C_Waves)	Bill Karsh (Janelia Research Campus)	https://billkarsh.github.io/SpikeGLX
Extracellular electrophysiology data processing pipeline	Jennifer Colonell (Janelia Research Campus)	https://github.com/jenniferColonell/ecephys_spike_sorting
Kilosort 2.5	Pachitariu et al., (2016)	https://github.com/MouseLand/Kilosort
Phy	Cyrille Rossant (International Brain Laboratory)	https://github.com/cortex-lab/phy
Shield-2018	Swaney et al., (2019)	https://hub.docker.com/r/chunglabmit/shield-2018 https://github.com/chunglabmit/shield-2018
Elastix	Klein et al., (2010)	https://elastix.lumc.nl
Lasagna	OpenSerialSection (University College London)	https://github.com/SainsburyWellcomeCentre/lasagna
Atlas Electrophysiology	International Brain Laboratory; Liu et al., (2021)	https://github.com/int-brain-lab/iblapps/tree/master/atlaselectrophysiology
AllenCCF	University College London	https://github.com/cortex-lab/allenCCF
Computation-through-dynamics (LFADS and fixed point analysis)	Pandarinath et al. (2018b); Sussillo and Barak (2013)	https://github.com/google-research/computation-thru-dynamics

Deposited data		

STARmap data	NeMO Archive	asset.nemoarchive.org/dat-9ACQ8G2
Electrophysiology data	DANDI Archive	N/A

## Highlights

- TH^+^ neurons in medial habenula encode reward-predictive cues
- Tac1^+^ neurons in medial habenula encode reward outcome and reward history
- Population dynamics of Tac1^+^ neurons track reward history via a putative line attractor
- Dynamical systems modeling predicts probability dependence of Tac1^+^ reward response
