# Supplementary material for: Cell-type-specific population dynamics of diverse reward computations
Source: Cell. Author manuscript; Available in PMC 2023 Jul 30. (PMC10387374; doi:10.1016/j.cell.2022.08.019)
Supplement: Table S1 [file NIHMS1907966-supplement-Table_S1.pdf]

Cell, Volume *185*

Supplemental information

Cell-type-specific population dynamics

# of diverse reward computations

Emily L. Sylwestrak, YoungJu Jo, Sam Vesuna, Xiao Wang, Blake Holcomb, Rebecca H. Tien, Doo Kyung Kim, Lief Fenno, Charu Ramakrishnan, William E. Allen, Ritchie Chen, Krishna V. Shenoy, David Sussillo, and Karl Deisseroth

Table S1. Statistical analysis of changes in fiber photometry fluorescence during 3CSRTT behavioral events, related to Figure 2.

		**Cue-Triggered Activity**		**Poke-Triggered Activity**	
**Genotype**	**Comparison**	**Pre-Cue**	**Post-Cue**	**Pre-Poke**	**Post-Poke**
TH	Correct vs Incorrect	0.36	0.42	0.32	*****<0.001**
	Correct vs Omitted	0.07	0.38	0.32	*****<0.001**
Tac1	Correct vs Incorrect	0.26	****<0.01**	***0.04**	*****<0.001**
	Correct vs Omitted	0.08	***0.03**	0.59	*****<0.001**
ChAT	Correct vs Incorrect	**0.01**	n/a	0.17	****<0.01**
	Correct vs Omitted	n/a	n/a	0.17	0.09
Calb1	Correct vs Incorrect	n/a	n/a	0.29	***0.01**
	Correct vs Omitted	n/a	n/a	0.29	0.19
LHb	Correct vs Incorrect	n/a	n/a	0.27	****<0.01**
	Correct vs Omitted	n/a	n/a	0.84	****<0.01**
