# Supplementary material for: Cell-type-specific population dynamics of diverse reward computations
Source: Cell. Author manuscript; Available in PMC 2023 Jul 30. (PMC10387374; doi:10.1016/j.cell.2022.08.019)
Supplement: 4 [file NIHMS1907966-supplement-4.pdf]

CellPress
OPEN ACCESS

Supplemental figures

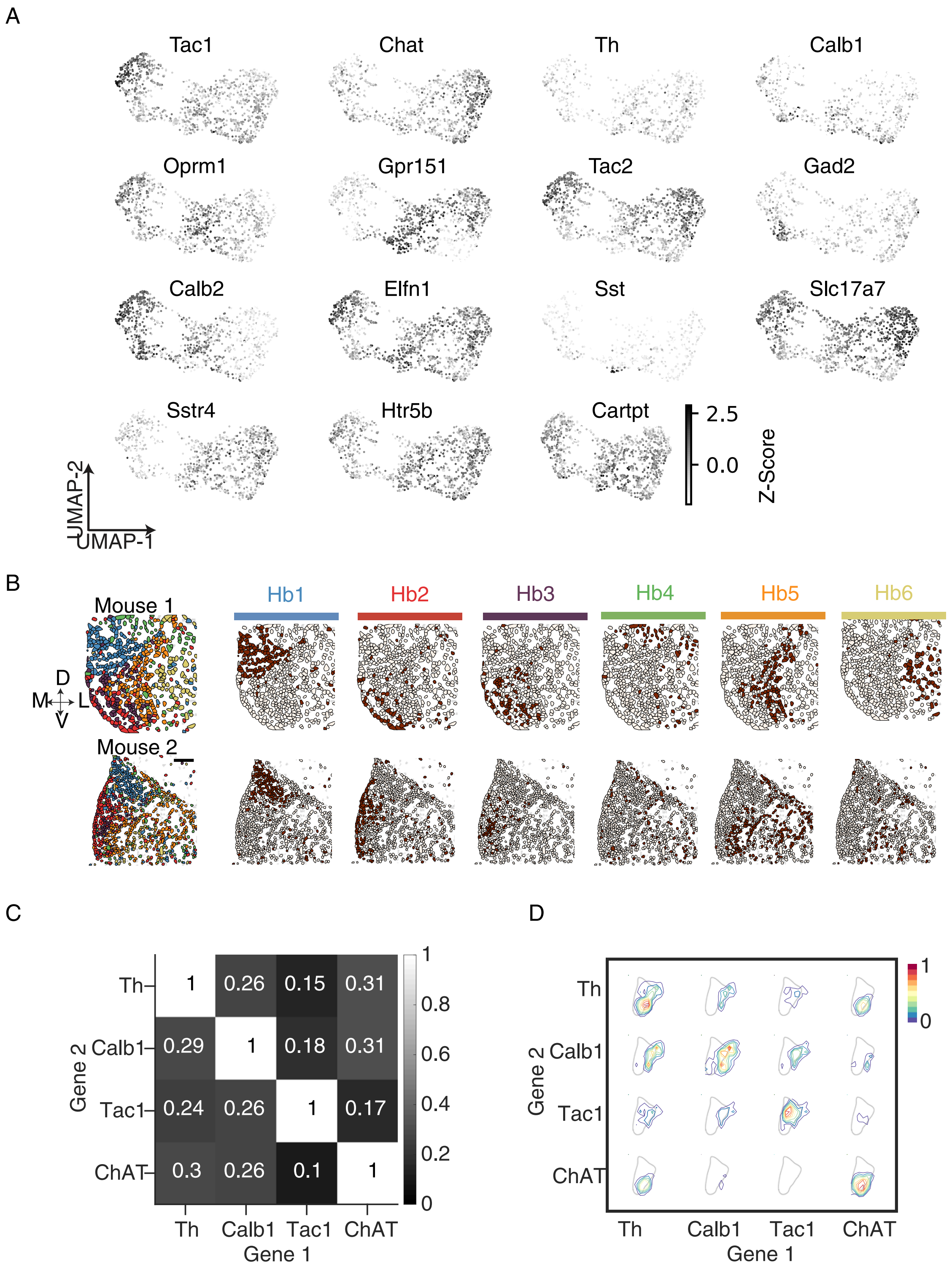

(legend on next page)

Figure S1. ***In situ*** **sequencing (STARmap) in habenular neurons, related to Figure 1**

(A) UMAP projections from STARmap *in situ* sequencing in Figure 1F. Grayscale indicates *Z* score(expression) for each gene.

(B) Spatial position of cells from each of the 6 clusters determined in Figure 1E. Scale Bar = 100 μm.

(C) Quantification of overlap of expression from STARmap for 4 genes also quantified by *in situ* hybridization in Figure 1I. Amplicon counts for each gene were Z scored and cells with *Z* score>0.5 were denoted as expressing that Gene. Grayscale indicates the proportion of cells expressing Gene 1 that also express Gene 2. Fractional overlap listed inside each box. n = 1440 neurons.

(D) Quantification of the spatial coexpression of genes from *in situ* hybridization in Figures 1H and 1I. Contours indicate the distribution of cells expressing Gene 1 that also express Gene 2, in 50 μm bins, normalized to the max expression of Gene 1. Diagonal represents spatial expression of individual genes.

CellPress
OPEN ACCESS

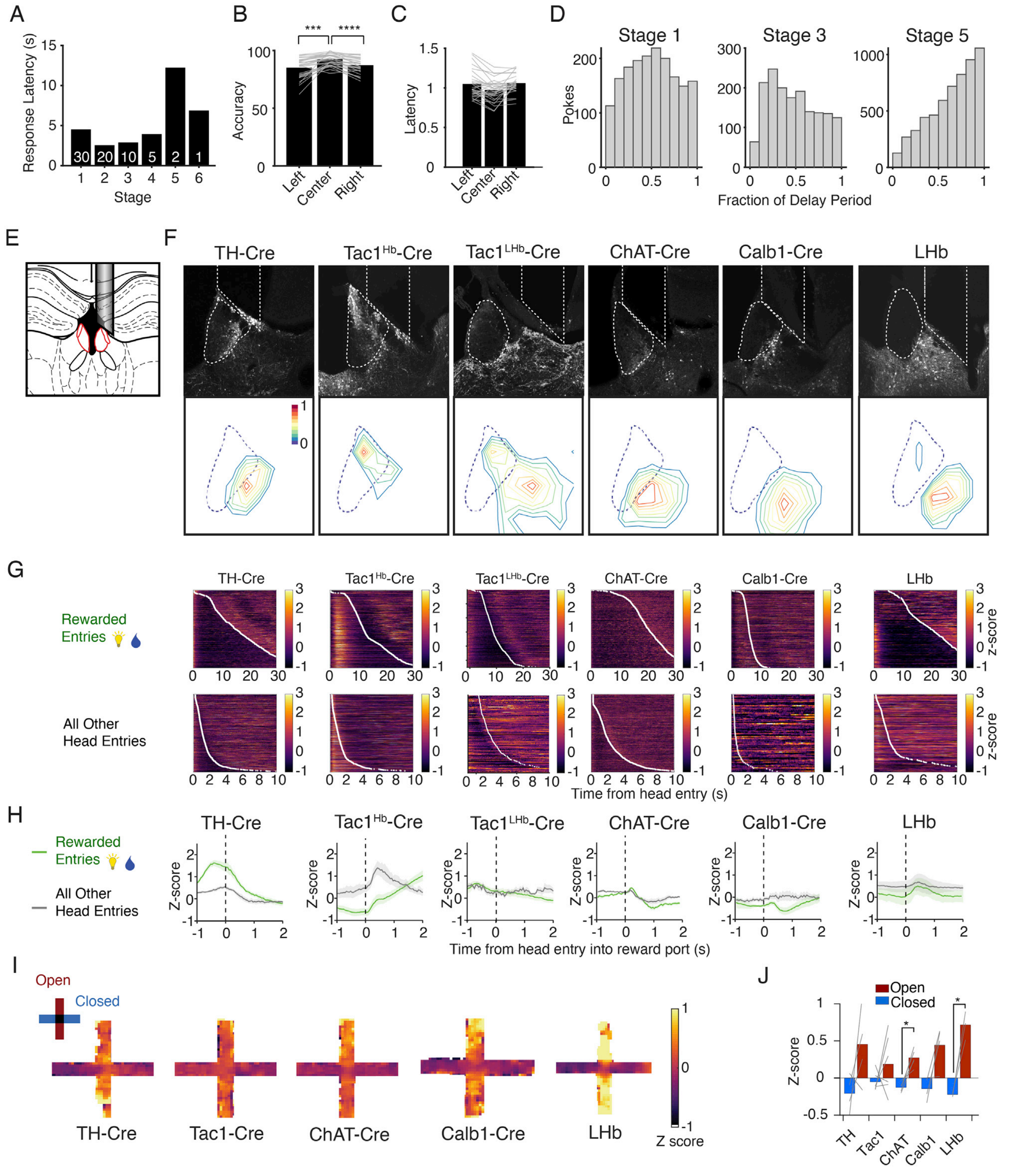

Figure S2. Neural activity of habenular neurons in freely moving behavior, related to Figure 2

(A) Average number of sessions necessary for an animal to advance to the next stage of training. White text indicates duration of cue light in seconds at each stage.

(B–C) Accuracy and latency for correct nose pokes as a function of nose poke location. Gray lines represent each animal. Latency, n.s.. Accuracy, Left vs Middle, ***P < 0.001; Right vs Middle ****P < 0.0001 by One-Way repeated measure ANOVA, FDR-corrected.

(legend continued on next page)

(D) Distribution of the latency of premature pokes at different stages of training. To account for variable delay durations, data is plotted as a fraction of the delay duration.

(E) Diagram indicating placement of optical fibers.

(F) Top, example GCaMP expression (AAV1-EF1a-DIO-GCaMP6f, immunostained with anti-GFP) and fiber placements for each genotype analyzed. Bottom, Quantification of spatial distribution of $GCaMP^+$ neurons within the Hb. Contours indicated the normalized cell density for each genotype. TH-Cre, n = 5; Tac1-Cre, n = 7; ChAT-Cre, n = 5; Calb1-Cre, n = 5. Scale bar = 200μm.

(G) Individual trials of rewarded and unrewarded head entries. Each row is an individual trial, sorted by the duration in the reward port and pseudocolored by *Z* scored fluorescence. t_0 = port entry. White dots indicate port exit for each trial. Tac1-Cre animals include those with AAV1 injections and fiber placements targeted to the MHb, which had an average of 82% of neurons in the MHb.

(H) Mean calcium fluorescence across genotypes at reward port entry. Green, rewarded port entries; Gray, all other entries during behavioral session (unrewarded). Error bars indicate SEM.

(I) Average *Z* score for each genotype as a function of position in the EPM. Color indicates *Z* score of fiber photometry fluorescence.

(J) Average *Z* score for closed arms and open arms. Gray lines indicate individual animals. ChAT, $p < 0.05$ closed vs open and center vs open. LHb, $p < 0.05$ closed vs open by One-Way ANOVA with repeated measures and FDR correction.

CellPress
OPEN ACCESS

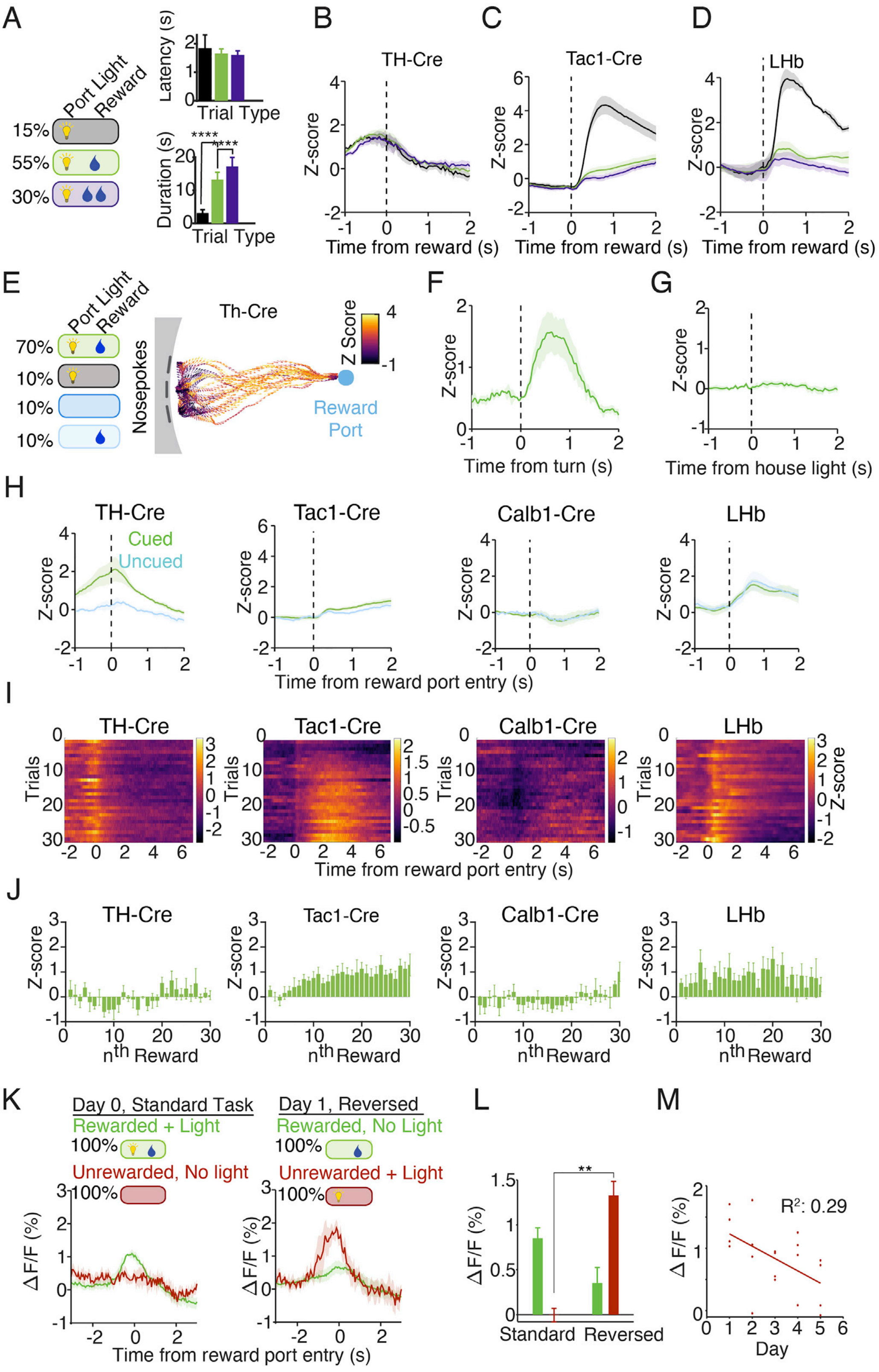

(legend on next page)

Figure S3. Cues and rewards modulate cell-type-specific activity in the habenula, related to Figure 3

(A) In another version of the 3-CSRTT, reward sizes were varied. Correct trials either resulted in a withheld reward (15% of trials, black), a 10µL reward (55% of trials, green) or a 20µL reward (30% of trials, purple).

(B–D) Reward-related activity in Hb cell types as a function reward size. Mean *Z* scored fluorescence data aligned to reward port entry and separated by reward volume. $Tac1^+$ neurons show decrease in activity at larger-than-expected rewards. Small rewards vs none: LHb, $p < 0.01$; Tac1, $p < 0.01$. Small vs large reward: LHb, $p = 0.1$; Tac1, $p = 0.03$. TH-Cre, n = 4 mice, 960 trials; Tac1-Cre, n = 7 mice, 3572 trials; LHb, n = 4 animals, 1270 trials. Tac1-Cre animals include those with AAV1 injections and fiber placements targeted to the MHb, which had an average of 82% of neurons in the MHb.

(E) Example behavioral tracking of a TH-Cre mouse in the 3CSRTT. Arrows indicate head direction in correct, rewarded trials, pseudocolored by the mean fluorescence for that video frame.

(F) *Z* scored fiber photometry data, aligned to time from the turn toward the reward port, calculated as the first frame where the head has swept to within 45° from the reward port.

(G) Fiber photometry data aligned to the illumination of the house light after time out from incorrect or premature trials.

(H) Activity of Hb cell types outside of trial-based operant training. Animals previously trained on the 3-Choice Task were given free rewards in sessions that contained no trial structure. Rewards were pseudorandomly delivered at 10–50s intervals. In 75% of trials, rewards were cued with the reward port light previously associated with reward, the remaining 25% were uncued. t = 0 represents head entry into reward port. Mean *Z* score of fluorescence aligned to reward delivery for cued and uncued rewards. Colors indicate cued (green) or uncued (light blue) rewards. Th^+, $p < 0.05$ by t test. Th-Cre, n = 5 mice; Tac1-Cre, n = 7 mice; ChAT-Cre, n = 5 mice; Calb1-Cre, n = 5 animals; LHb, n = 4 animals. Tac1-Cre animals include those with AAV1 injections and fiber placements targeted to the MHb, which had an average of 82% of neurons in the MHb.

(I) Mean change in reward response over the course of a behavioral session with randomly delivered rewards, as in H. Each row indicates trial number, *Z* scored across the session, averaged across all animals of each genotype. Both cued and uncued rewards are shown.

(J) Mean reward response over the session, calculated at the mean 0–4 s after reward minus the mean 1s before entry to the reward port. Error bars indicate SEM.

(K) Reversal paradigm for reward-guided behavior in trained TH-Cre animals. Predictive cues and outcomes were switched: incorrect and premature trials (red) were associated with a reward port light whereas correct trials (green) were not. Animals were trained on the reversal paradigm for 5 sessions. Mean photometry signal for the first reversal session, *Z* scored and aligned to reward port entry. Error bars indicate SEM.

(L) Mean change in *Z* score of fluorescence on the last day of standard training (Day 0) and the first day of a reversed light contingency (Day 1). Fluorescence calculated using the mean *Z* score 500ms prior to reward port entry. Error bars indicate SEM n = 4 animals.

(M) Mean change in *Z* score of fluorescence calculated using the mean *Z* score 500ms prior to reward port entry during each of five days of reversal training. Error bars indicate SEM n = 4 animals. $R^2 = 0.29$; $p < 0.05$.

CellPress
OPEN ACCESS

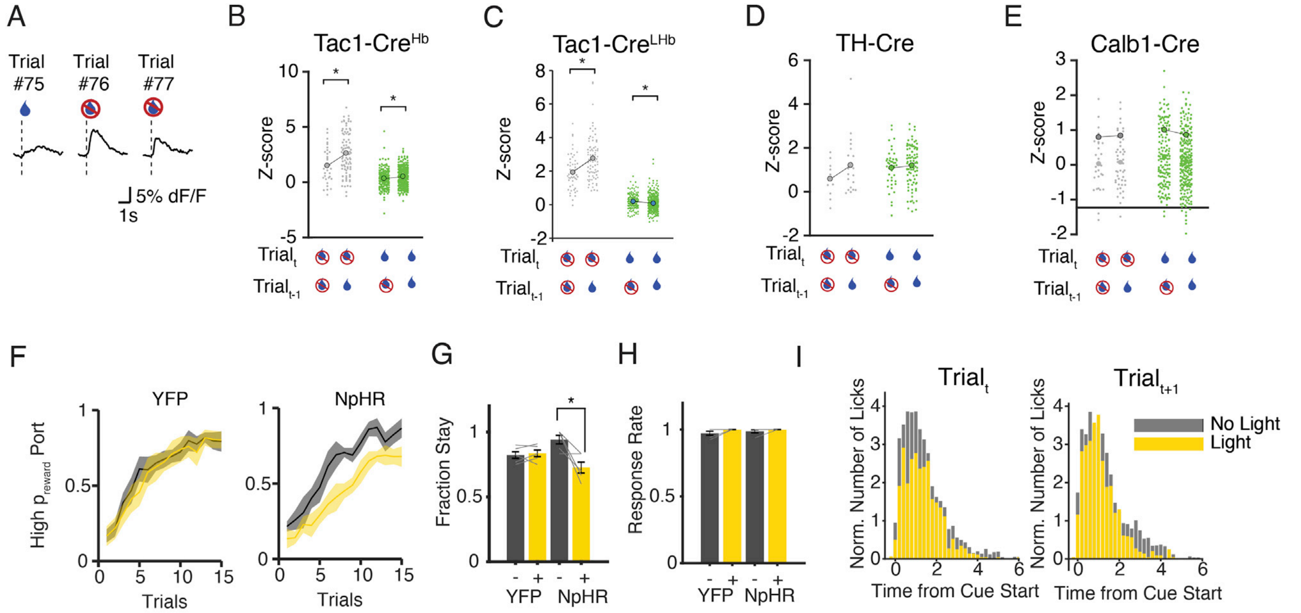

Figure S4. Recent trial history modulates habenular activity, related to Figure 4

(A) Example of three consecutive trials demonstrating rewarded::withheld (#75/76) and withheld::withheld (#76/77) trial histories.

(B–E) Change in fluorescence as a function of previous trial history for four Hb cell types from data in Figures 3A–3F, 80% of trials rewarded, 20% withheld. Each data point indicates the *Z* score for a delivered reward (green) or a withheld reward (gray), separated by the reward availability of the previous trial. Mean *Z* score is calculated during the time window corresponding to the full width at half max. Tac1, $p < 0.01$ current trial unrewarded, $p = 0.02$, current trial rewarded. $Tac1^{Hb}$, n = 7 mice, 2860 trials; $Tac1^{LH}$, n = 7 mice, 5840 trials; TH, n = 4 mice, 970 trials; Calb1, n = 5 animals, 2240 trials. $Tac1\text{-}Cre^{Hb}$ animals include those with AAV1 injections and fiber placements targeted to the MHb, which had an average of 82% of neurons in the MHb. $Tac1\text{-}Cre^{LHb}$ animals include those with serotype targeted AAV8 injections and fiber placements targeted to the MHb, which had an average of 88% of neurons in the LHb.

(F) Fraction of choices to the new (high reward) port in the head-fixed reward-guided decision-making task for YFP control and $Cre^{On}Flp^{Off}$ eNpHR3.0 Tac1-Cre animals. $Trial_0$ is the last trial of the previous block. Error bars indicate SEM.

(G) For any rewarded $trail_t$ at the block switch (trials 1–15 from switch), the fraction of $trial_{t+1}$ in which the animal licks at the same port as $trial_t$ is calculated (win-stay fraction).

(H) Response rate for $trial_{t+1}$ if $trial_t$ was an inhibited trial or a control trial.

(I) Lick distribution in reward-guided decision-making task for trials with or without illumination, normalized to the total number of licks. All error bars indicate SEM.

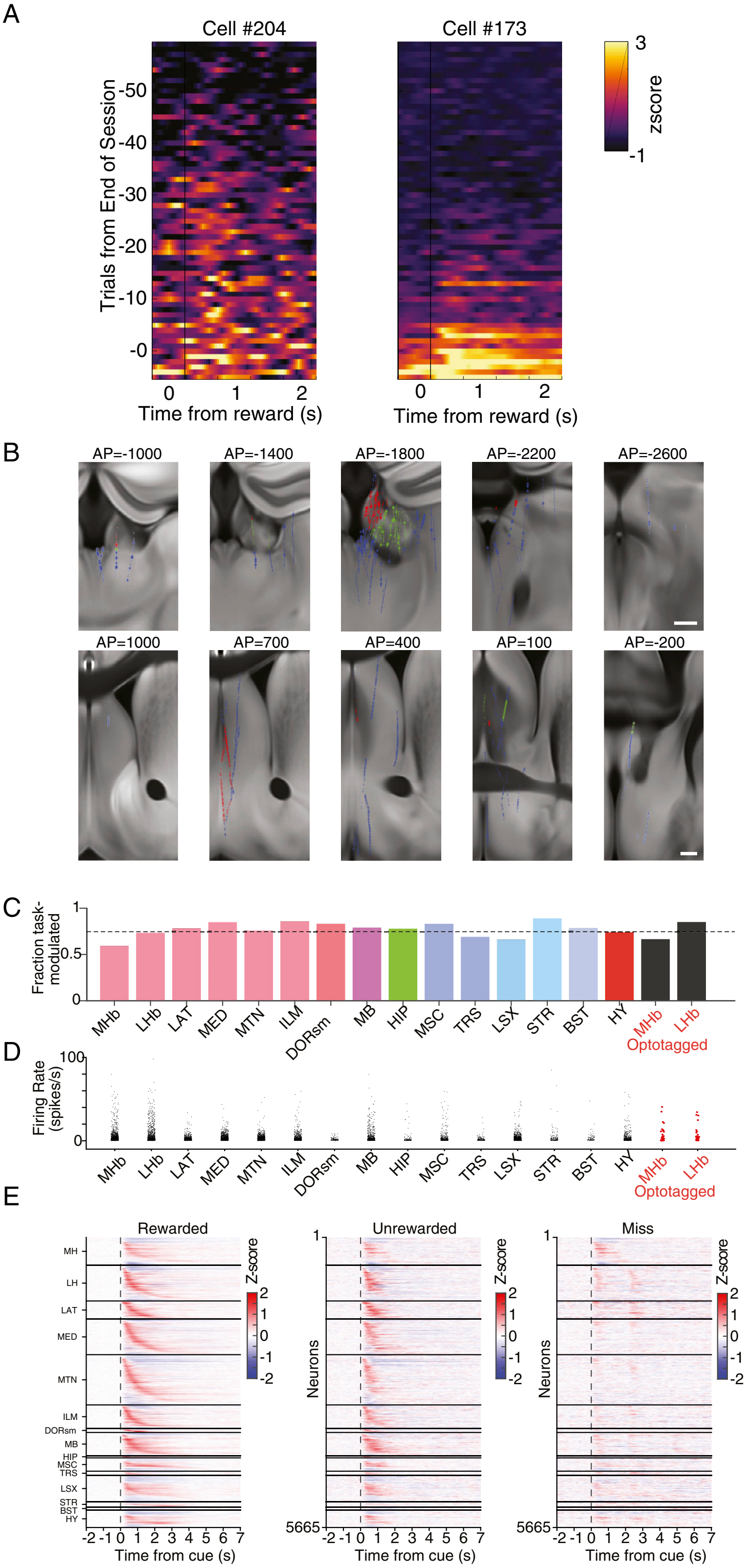

(legend on next page)

CellPress
OPEN ACCESS

Figure S5. Extended data on cellular-resolution imaging and electrophysiology, related to Figure 5

(A) Reward responses of two example Tac1$^+$ neurons across a behavioral session. Left, a ramping neuron (R^2 = 0.65). Right, a sigmoid-accumulating neuron (R^2 = 0.57).

(B) Top, Spatial position of recorded single neurons registered to the Allen CCFv3 atlas, targeting MHb. Red, MHb; green, LHb; blue, others; *, optotagged. Bottom, Spatial position of recorded single neurons registered to the atlas, targeting septum. Red, MSC; green, TRS; blue, others. N = 2181 septal neurons from 10 mice in 10 sessions.

(C) Fraction of task-modulated neurons across regions. See Methods for quantification.

(D) Average firing rate across regions.

(E) Summary of trial-averaged, baseline-subtracted single-neuron activity across regions. Single-neuron activity was sorted within each region based on peak time for rewarded trials. Order of neurons are identical across trial types.

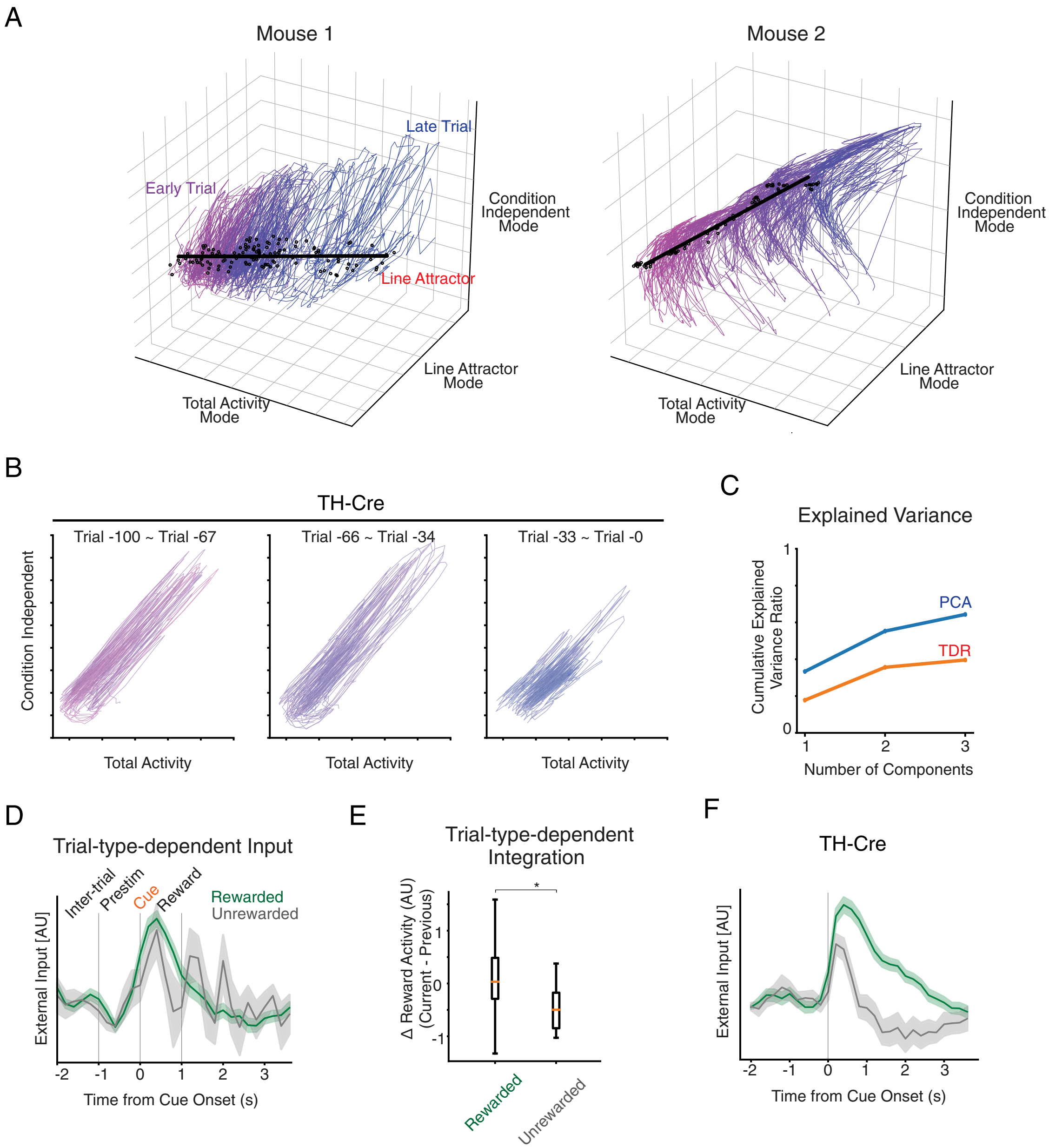

Figure S6. Extended data on the state space analysis, related to Figure 6

(A) All single-trial trajectories and fixed points for Tac1$^+$ trLFADS models for mouse #1 and #2. The three axes, identified by a targeted dimensionality reduction approach, were orthonormalized. Colored dots indicate slow points found by fixed point analysis. The line is the top PC through the space of slow points. Note the presence of the line attractor dynamics in both mice and that the total activity mode and the line attractor is highly aligned. The rest of the analyses with Tac1$^+$ neurons are visualized with mouse #1.

(B) Sets of single trial trajectories for TH$^+$ trLFADS model for mouse #1. The trials were grouped in time to highlight the absence of the activity accumulation feature in Tac1$^+$ mice. The rest of the analyses with the trLFADS model for TH1$^+$ neurons is visualized with mouse #1.

(C) Explained variance in the state space plots for the trLFADS models for Tac1$^+$ neurons. The ordered components from targeted dimensionality reduction are the total activity mode, the condition independent mode, and line attractor mode.

(D) Single-trial inferred external input for Tac1$^+$ trLFADS model shows trial-type-dependent temporal profile, specifically after the cue onset. Mean $^+/-$ SEM.

(E) The distinct inferred external input for Tac1$^+$ trLFADS model results in larger shifts of total activity in rewarded trials than in unrewarded trials, implying trial-type-dependent integration of reward history. Wilcoxon rank-sum test; *p < 0.01.

(F) Single-trial external inputs for TH$^+$ neurons also show trial-type-dependent temporal profile, although these inputs are not integrated due to the discrete fixed point arrangement.

CellPress
OPEN ACCESS

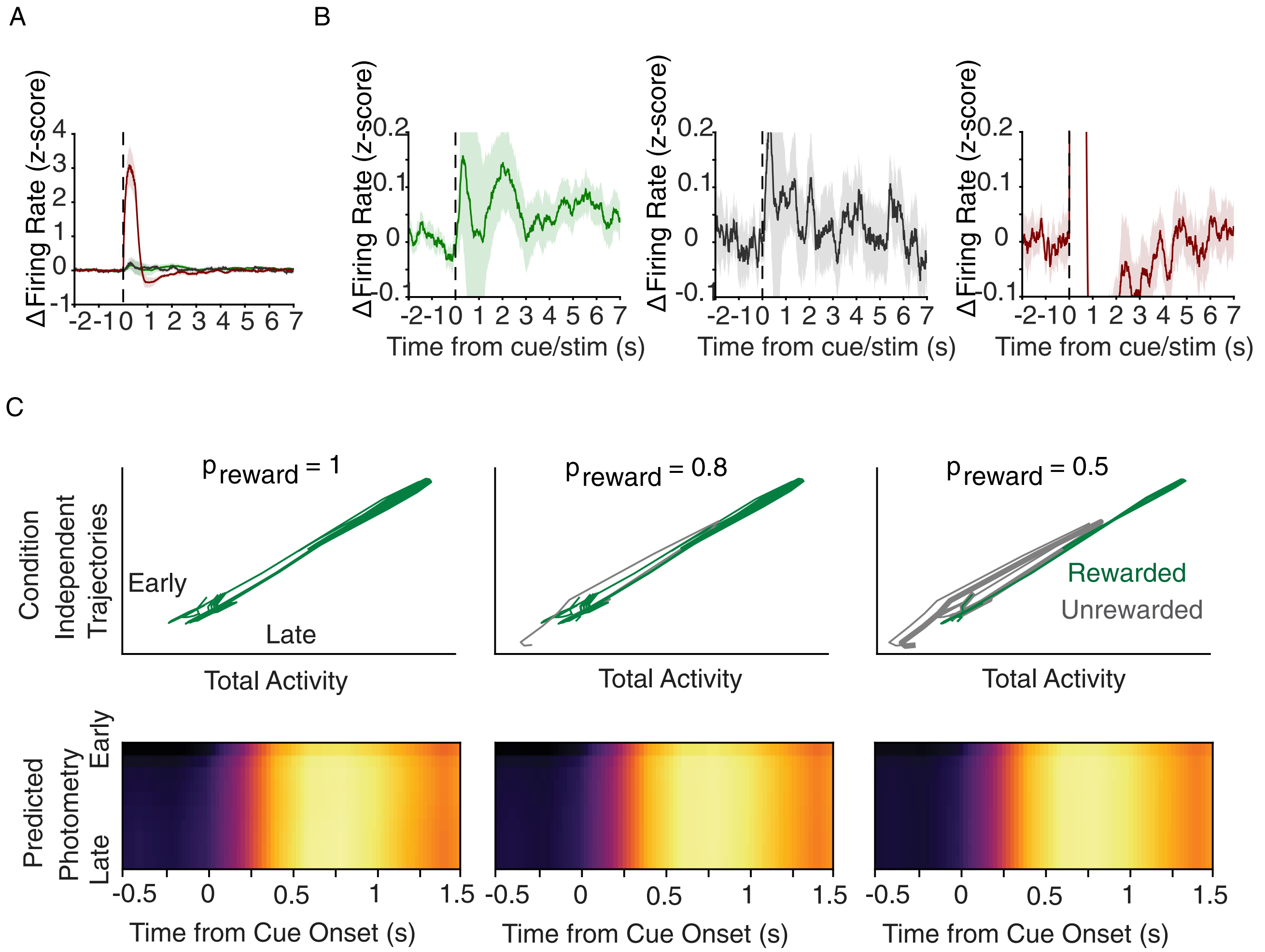

Figure S7. Extended data on model-guided experiments, related to Figure 7

(A) Average firing changes in rewarded (green), unrewarded (black), and perturbation (red) trials. Curves, mean; shaded error, SEM from hierarchical bootstrap.

(B) Zoomed-in visualization of (A) for each trial type. 2 s windows were used for baseline subtraction (−2 to 0 s) and within-trial firing rate change quantification (−2 to 0 s; 5 to 7 s).

(C) In silico dynamics simulation of TH^+ neurons predicts no reward history accumulation regardless of the reward probability, as expected.
